# Quality of medical products for diabetes management: a systematic review

**DOI:** 10.1136/bmjgh-2019-001636

**Published:** 2019-09-24

**Authors:** Kartika Saraswati, Chanvilay Sichanh, Paul N Newton, Céline Caillet

**Affiliations:** 1 Lao-Oxford-Mahosot-Wellcome Trust Research Unit, Microbiology Laboratory, Mahosot Hospital, Vientiane, Lao People's Democratic Republic; 2 Centre for Tropical Medicine and Global Health, Nuffield Department of Medicine, University of Oxford, Oxford, UK; 3 Infectious Diseases Data Observatory/WorldWide Antimalarial Resistance Network, Centre for Tropical Medicine and Global Health, University of Oxford, Oxford, UK

**Keywords:** medicine quality, substandard and falsified medicine, diabetes

## Abstract

**Background:**

The global prevalence of diabetes mellitus is increasing alarmingly. However, the quality of vital medicines and medical products used to treat and monitor diabetes remains uncertain but of potential great public health significance. Here, we review the available evidence on the quality of antidiabetic medicines and supplies for self-monitoring of blood glucose (SMBG) and discuss their potential impact for the patients and society.

**Methods:**

Searches were conducted in PubMed, Embase, Google Scholar, Google and relevant websites in English and French. The Medicine Quality Assessment Reporting Guideline (MEDQUARG) was used to assess the quality of medicine quality surveys.

**Results:**

52 publications on the quality of antidiabetic medicines, including 5 medicine quality prevalence surveys and 20 equivalence studies, were analysed. The prevalence surveys and equivalence studies included 674 samples of which 73 (10.8%) were of poor quality. The median (Q1–Q3) concordance with MEDQUARG items was 30.8% (19.2%–42.3%). No prevalence surveys on SMBG supplies’ quality were found, but 29 publications, including falsified products and incorrect results due to strip degradation or contamination, were identified.

**Conclusion:**

There is little accessible evidence on the quality of antidiabetic medicines and SMBG supplies. Surveys were poorly designed and reported, making data aggregation and interpretation problematic. Despite these caveats, these results suggest that there are important issues with the quality of medical products for diabetes that need focused monitoring. There is an urgent need to achieve consensus protocols for designing, conducting and reporting medical product quality surveys.

**PROSPERO registration number:**

CRD42016039841.

**Key questions**

**What is already known?**

- Substandard and falsified medical products for managing diabetes will negate the benefits of modern diabetes treatment and the public benefit of reduced complications.
- Better understanding of the epidemiology of substandard and falsified diabetes medications and self-monitoring of blood glucose (SMBG) supplies and their potential impact on patients and society is needed.

**What are the new findings?**

- Our study demonstrated that of 674 samples analysed, about 1/10 were substandard or falsified but this prevalence is not generalisable globally.
- Issues with the quality of SMBG supplies were identified (including falsified products and incorrect results due to strip degradation or contamination).

**What do the new findings imply?**

- Our findings showed that, despite the scarcity of the data, there are important issues with the quality of medical products for diabetes that need to be tackled.
- Besides increasing the quality of the surveys, consensus would facilitate evidence pooling and summarising to better understand the global burden of this problem.

## Introduction

Diabetes mellitus (hereafter diabetes) is a growing threat to global health with significant mortality and morbidity related to myriads of non-communicable and communicable disease consequences. In 2015, diabetes afflicted ~415 million people globally and this is predicted to reach 642 million by 2040, equivalent to 1 in 10 adults.^1^ The direct annual global cost of diabetes is estimated at US$827 billion.^2^

In the USA, approximately four in five adults with diabetes rely on antidiabetic medicines to control their glucose levels.^3^ Insulin is required for type 1 diabetes treatment, and is also used widely for advanced type 2 diabetes. In 2015, insulin was within the top 10 best-selling medications globally (in terms of number of prescriptions and sales value).^4 5^ It is estimated that globally ~100 million people need insulin.^6^ Type 2 diabetes treatment involves lifestyle change encouragement, but antidiabetic medicines are often required for the control of hyperglycaemia and prevention of long-term complications.^1 7 8^ Oral antidiabetic sale in 2016 was estimated to reach close to US$20 billion.^9^ There has also been a surge in innovative expensive oral therapies such as gliptins.

There are key public health issues with cost and access to antidiabetic medicines, especially insulin,^5^ and to specialised diabetes care. With huge price variation between countries and increasing demand, insulin and new oral antidiabetic medicines are likely to be attractive targets for falsification and are at risk of substandard production.^10^ High prices of some antidiabetics may prompt patients to look for more affordable options, including illegitimate sources such as unregistered physical and internet pharmacies.^11^

Nearly 60% of the global costs of diabetes are borne by low-income and middle-income countries (LMICs), with substantial treatment costs paid out-of-pocket and insufficient medicines regulation. This limits access and thus risks use of poor quality medical products, exacerbating financial hardship and impairing peoples’ lives and productivity.^12^

Patients with diabetes often need to check their blood glucose concentration multiple times per day using strip-based hand-held glucose meters. The cost for such self-monitoring of blood glucose (SMBG) can account for approximately one-third of the total cost of consumables, including insulin and needles, for type 1 diabetes management.^13^ This expense has prompted both falsification of glucose strips and reselling of secondhand unused glucose strips at reduced price.^10 14 15^ In 2008, the US Food and Drug Administration (FDA) and the American Diabetes Association warned against using resold strips as they may give incorrect results.^14 16^

Due to their vital role in diabetes management, poor quality antidiabetic medicines and SMBG supplies will inevitably have adverse health impacts for patients, both short and long term. Low active pharmaceutical ingredient (API) content and, for oral preparations impaired gastrointestinal dissolution, will increase the incidence of macrovascular and microvascular complications due to compromised glucose control, and hence increase individual and societal economic costs.^17–19^ High API content may also bring grim immediate consequences. Deaths have been linked to falsified antidiabetic medicines in China containing dangerously high amounts of the oral antidiabetic agent glibenclamide.^20 21^ Poor quality SMBG supplies will impair patients with diabetes from receiving correct medication doses, potentially causing life-threatening hypoglycaemia, hyperglycaemia and long-term consequences. Poor quality antidiabetics and SMBG supplies will also decrease faith in the health system, confuse patient and clinical decision-making and risk use of unapproved treatments.

The circulation of poor quality medicines and medical products, whether falsified, substandard or degraded, is a major global public health problem. No country is immune.^22–28^ A recent WHO report suggested that in LMICs, ~10.5% of medicine samples (mainly anti-infectives) analysed were substandard or falsified (SorF).^28^ Although medical product regulatory systems are usually reliable and functional in high-income countries, poor quality medicines and medical devices have been identified.^11 15 27 29^ The extent of the problem seems more pronounced in financially poor countries where regulatory systems are often weak.^24 26 27 30^ Rising concern about the consequences of poor quality medicines over the last decade has focused on anti-infectives.^31^ However, there is growing awareness of this issue for chronic non-communicable diseases, for example, a recent survey described that 16.3% of cardiovascular medicines sampled in ten sub-Saharan African countries were poor quality.^32^ These will have significant public health impact and if poor quality diabetes medical products are also prevalent they will have an additive global toll on health. The recent WHO report stated that seven member states reported 11 substandard and falsified (SF) diabetes medicines between 2013 and 2017 but details are not given.^28^ As there is no global understanding of the epidemiology of poor quality medical products for diabetes, we reviewed the available evidence and discuss the potential impact for patients and society.

## Methods

### Search strategy

Reports were identified through systematic searches in PubMed, Embase, Google Scholar in English and French up to 30 April 2018, using the search terms (online supplementary file 1) ‘diabetes’, ‘antidiabetics’, the names of active pharmaceutical ingredients (eg, ‘insulin’, ‘metformin’, ‘glibenclamide’), ‘glucose meter’, ‘strip’; combined with terms relevant to medicine quality (eg, ‘falsified’, ‘counterfeit’, ‘substandard’, ‘degraded’). Reports were also identified in Google from the first 20 pages of results and other sources such as websites of international organisations interested in medicine quality and medicines regulatory authorities (online supplementary file 1). After removal of duplicates, the titles and abstracts were first screened, and full text of the identified articles were then assessed for eligibility. The reference lists of the eligible articles were also manually screened for inclusion.

10.1136/bmjgh-2019-001636.supp1 Supplementary data

### Eligibility criteria

Published scientific articles and grey literature assessing or discussing the quality of antidiabetic medications were included. Articles describing the development or assessing the performance of chemical techniques for the analysis of antidiabetics quality were excluded. Articles without clear conclusion on the quality of the assessed products or with uninterpretable results (eg, conflicting quality results for the same samples analysed in two different laboratories)^33^ were excluded. Articles on the quality of SMBG supplies found through the searches were included. However, articles describing the accuracy and performance of glucose meters and strips were excluded, as were reports on the quality of insulin syringes, pens and needles.

### Key definitions

‘Falsified’ refers to products that ‘deliberately/fraudulently misrepresent their identity, composition or source’.^28 30 34^ In this review, ‘fake’, ‘counterfeit’, ‘spurious’ and ‘falsely labelled’ medicines are regarded as synonyms of falsified, a term that emphasises public health rather than intellectual property issues inherent in the term ‘counterfeit’. ‘Substandard’ medicines, also called ‘out of specification’, are authorised medical products that ‘fail to meet either their quality standards or their specifications, or both’.^34^ This may result from negligence or errors during the manufacturing process by authorised manufacturers,^28 35^ or degradation through deterioration because of inappropriate storage/transport in the supply chain.^11^ Information is usually insufficient to distinguish errors within factories from those in the supply chain, a key evidence gap as the solutions for the two differ.^22^ As it is not possible to reliably classify a medicine as substandard or falsified without packaging analysis,^22^ in this review products that failed at least one quality test without information on packaging authenticity, and falling outside the acceptance range of the specifications chosen as reference by the authors (either specific pharmacopoeia monograph or in-house specifications), are defined as ‘substandard or falsified’ (SorF).

‘Prevalence surveys’ are studies in which samples were collected within the pharmaceutical supply chain to assess their quality, in order to describe the prevalence of circulating SF medicines. ‘Equivalence studies’ are those whose main objective is to assess the quality of different marketed brands of the same API(s) assuming that the collected samples would represent the quality of the brand and not an estimate of the frequency of individual samples of different quality. In most cases, equivalence studies aim at providing information on brand(s) as a whole, whereas prevalence surveys aim at evaluating individual samples of marketed medicines to give an estimate of their frequency in a community.

### Risk of bias assessment

The prevalence surveys methodology and reporting were assessed using Medicine Quality Assessment Reporting Guidelines (MEDQUARG). MEDQUARG is a comprehensive checklist composed of 26 items proposed to be included in the reporting of medicine quality surveys.^36^ For each item, all criteria are to be fulfilled to be awarded one point. Prevalence surveys were assessed independently by two investigators with a third person resolving any disagreement. Since there were no standardised tools available to assess lay types of publication, their risk of bias was not specifically assessed.

### Data collection, analysis and reporting

The data extracted included year of publication, publication type, definition used for medicine quality, location of survey, sampling strategy, sample size and failure rate (with additional description of the type of failure when applicable). A ‘data point’ is defined as a location where medicines were collected for quality analysis, at a given time and during a given study. A Microsoft Access database 2013 used in a similar review from our group was adapted for the purpose of the study.^22^ Microsoft Excel 2013 and RStudio V.0.99.486 were used for the data analysis. Qualitative variables were expressed as numbers and percentages (n (%)). Quantitative variables were expressed as median, as well as first and third quartiles (Q1 and Q3, respectively).

This review is reported according to the Preferred Reporting Items for Systematic Reviews and Meta-Analyses guidelines (online supplementary file 2).^37^

10.1136/bmjgh-2019-001636.supp2 Supplementary data

### Patient and public involvement

This research was done without patient or public involvement.

## Results

**Figure 1:**
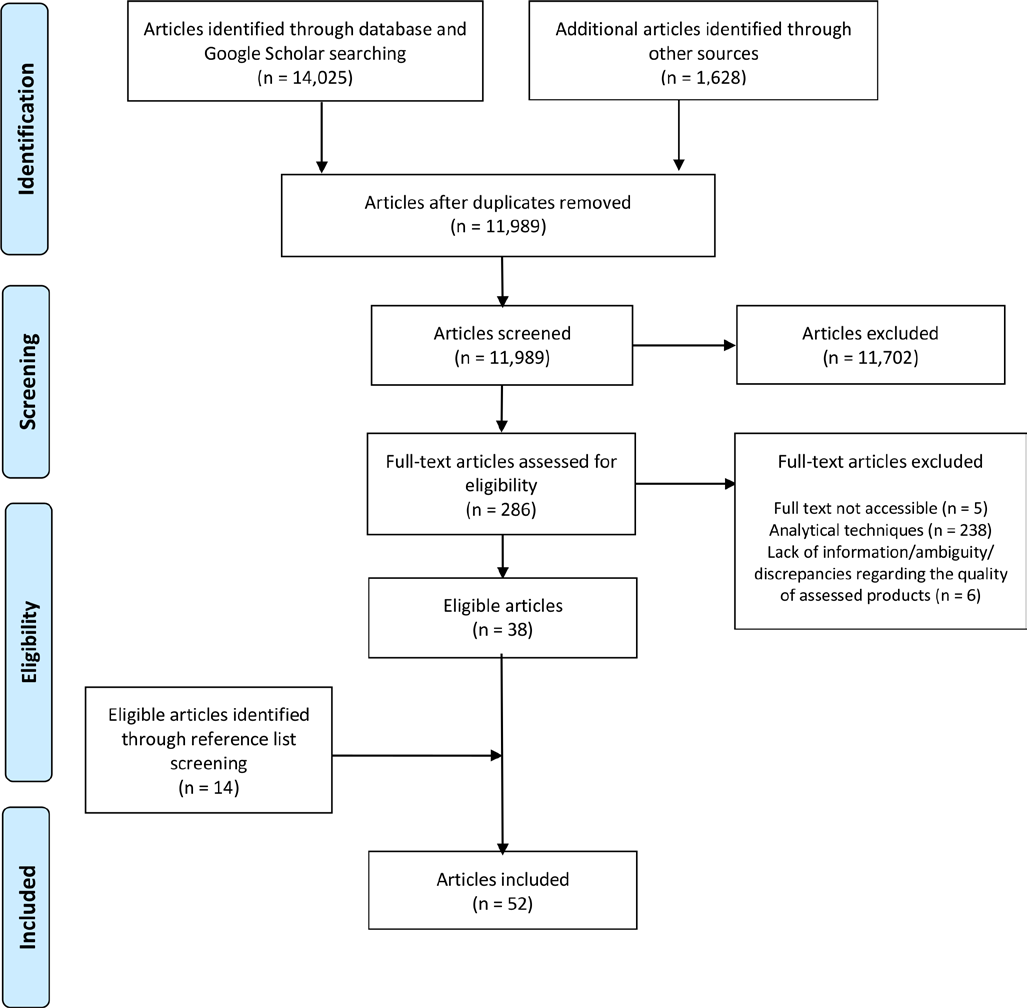
PRISMA flow diagram of the selection process of the publications on antidiabetic medicine quality. PRISMA, Preferred Reporting Items for Systematic Reviews and Meta-Analyses.

After removal of duplicates, 11 989 out of 15 653 publications gathered through electronic searches and other sources identified in the methods were screened by title and abstract (figure 1).

**Figure 2:**
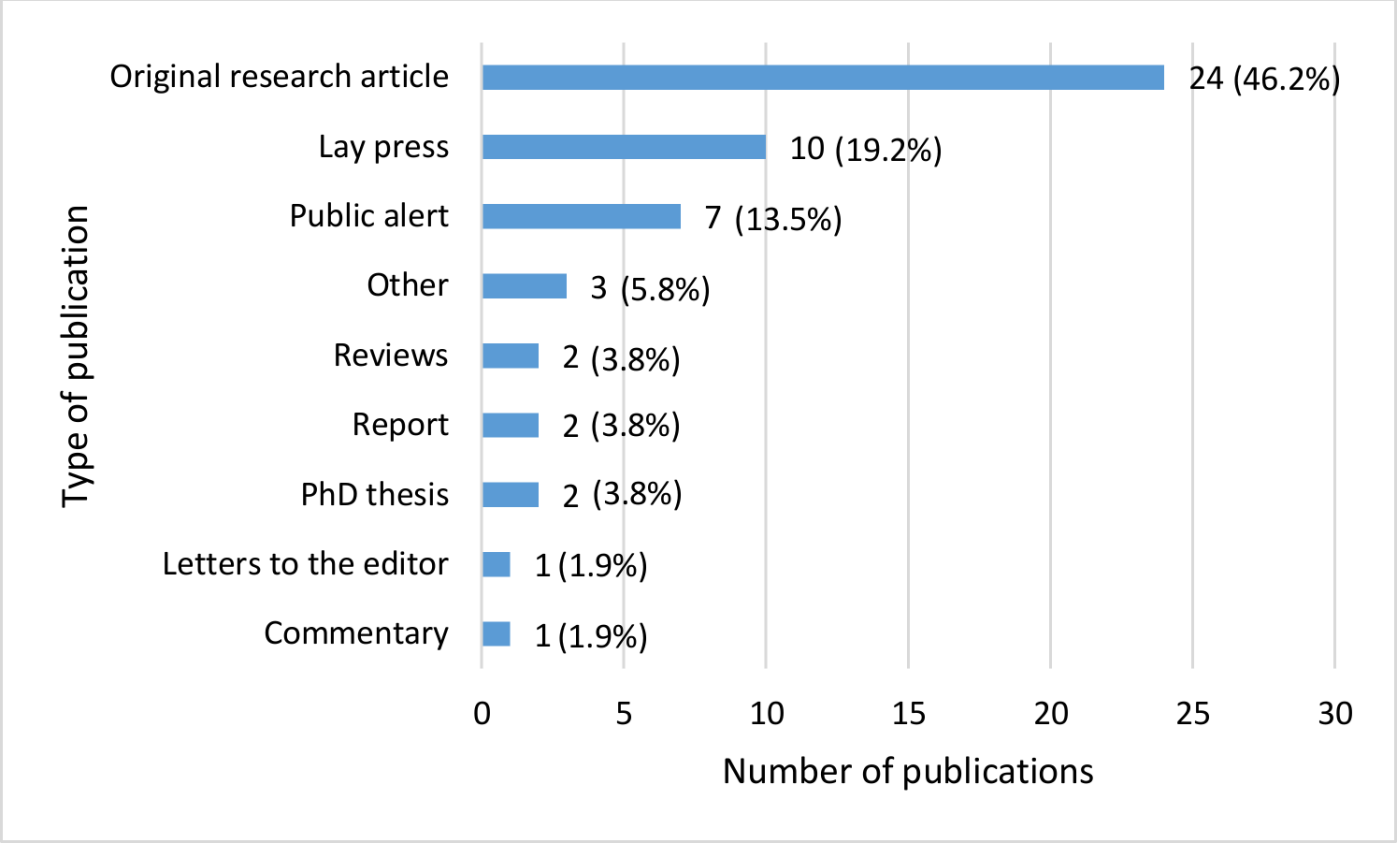
Types of publications related to the quality of medicines for diabetes.

**Figure 3:**
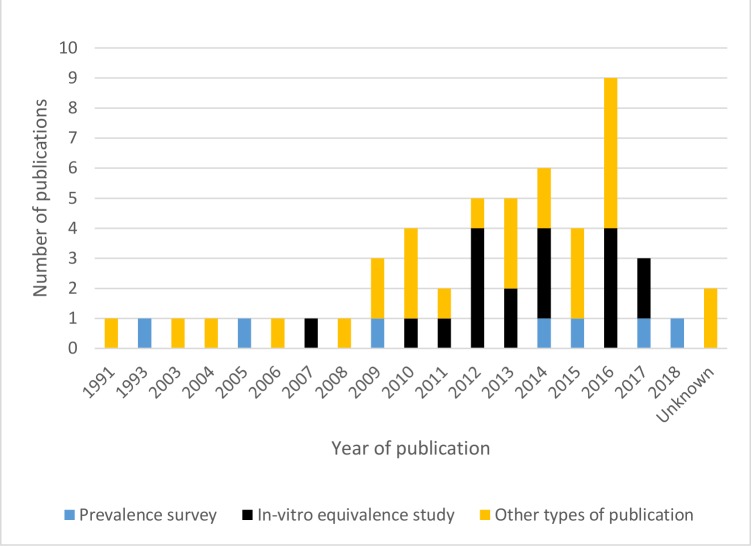
Number of publications over time related to the quality of medicines for diabetes.

Of these, 286 full-text publications were reviewed and 38 were included. Fourteen additional publications identified through manual reference screening were also included, yielding a total of 52 publications. Twenty-four (46.2%) of the included publications were original research articles published in scientific journals (figure 2). The number of publications increased from one publication in 2008 to nine in 2016 but decreased to three in 2017 (figure 3). Two publications (3.8%) had unstated year of publication. All publications will be mapped on the Infectious Diseases Data Observatory Medicine Quality Surveyor system (www.iddo.org).

Out of the 52 publications, 5 were prevalence surveys (9.6%) and 20 (38.5%) were equivalence studies (table 1). All surveys and studies included evaluated oral antidiabetics. The most commonly assessed are metformin (in four prevalence surveys and 13 equivalence studies) and glibenclamide (in two prevalence surveys and five equivalence studies). We found one survey investigating the quality of 84 insulin samples in 1979.^38^ However, due to the absence of reference to the acceptance range of the specifications chosen for the ‘conventional insulin preparations’ at the time of the study, we decided to exclude this article from this review.

**Table 1:** Main characteristics of prevalence surveys and equivalence studies of antidiabetic medicines included in the reviewBecause of the limited number of samples tested for quality in the studies included in this review, the numbers should not be interpreted as representative of the prevalence of specific SF antidiabetics (please refer to the discussion section of the current paper for more details)

Study	Country	Active pharmaceutical ingredient	Total no of samples collected	Failed samples n (%)
**Prevalence surveys**				
Blume et al^85^	Argentina, Australia, Austria, Belgium, Canada, Chile, Commonwealth of Independent States, Denmark, Egypt, Finland, France, Germany, Greece, Hungary, Indonesia, Italy, Japan, Luxembourg, Netherlands, Pakistan, Portugal, Spain, Sweden, Switzerland, Thailand, Turkey, UK, USA	Glibenclamide	187	8 (4.3)
Westenberger *et al* 40	Unstated	Metformin	4	0 (0.0)
Central Drugs Standard Control Organisation^86^	India	Metformin, gliclazide, glimepiride	45	0 (0.0)
Ebenezer^87^	Nigeria	Metformin	179	7 (3.9)
Islam^39^	Cambodia	Metformin, glibenclamide	112	21 (18.8)*
**Equivalence studies**				
Attorrese and Massi-Benedetti^70^	Unstated	Glimepiride	23	12 (52.2)
Hamdan and Jaber^88^	Jordan	Metformin	5	1 (20.0)
Chandrasekaran *et al* 89	Malaysia	Metformin	5	0 (0.0)
Afifi and Ahmadeen^90^	Saudi Arabia	Metformin	6	0 (0.0)
Chatur et al^91^	Unstated	Voglibose	5	1 (20.0)
Olusola *et al* 92	Nigeria	Metformin	8	1 (12.5)
Oyetunde *et al* 93	Nigeria	Metformin	5	2 (40.0)
El-Sabawi *et al* 94	Jordan	Glibenclamide	6	3 (50.0)
Labu *et al* 95	Bangladesh	Metformin	7	0 (0.0)
Ajala *et al* 96	Nigeria	Metformin	8	3 (37.5)
Betari and Haidar^97^	Unstated	Sitagliptin	5	2 (40.0)
Elango and Shanmuganathan^98^	India	Metformin	15	3 (20.0)
Elhamili *et al* 99	Libya	Glibenclamide	3	0 (0.0)
Abdulhameed *et al* 100	Iraq	Metformin	5	0 (0.0)
Gupta *et al* 101	Trinidad and Tobago	Metformin	4	0 (0.0)
Sachan *et al* 102	India	Metformin	4	0 (0.0)
Sakr *et al* 103	Saudi Arabia	Glibenclamide	8	0 (0.0)
Alam *et al* 104	Saudi Arabia	Glibenclamide	5	0 (0.0)
Eraga *et al* 105	Nigeria	Metformin	10	8 (80.0)†
Aivalli *et al* 106	India	Metformin, glibenclamide	10	0 (0.0)

*In Islam 2017,^39^ only the number of medicine failing each quality test was mentioned. Since one medicine may fail more than one test, the failure rate was recorded as the highest possible number of samples failing one of the tests.

†In Eraga 2017,^105^ uniformity of content was assessed using two methods that is, UV spectrophotometry and reverse-phase high-performance liquid chromatography. There are several discrepancies in the results of these two tests. Therefore, if samples failed either, they will be categorised as failed samples.

SF, substandard and falsified; UV, ultraviolet.

High-performance liquid chromatography (HPLC) and thin-layer chromatography analyses were conducted in four and one prevalence surveys, respectively. Various technologies were used in equivalence studies such as HPLC or UV-visible spectrophotometry (online supplementary file 3). The British Pharmacopoeia and United States Pharmacopeia (USP) were the most commonly followed standards (in 12 and 11 articles, respectively).

10.1136/bmjgh-2019-001636.supp3 Supplementary data

In total, 674 samples were collected in 38 countries; 527 (78.2%) from 31 countries for prevalence surveys and 147 (21.8%) from 9 countries in equivalence studies (table 2). The median (IQR) number of samples collected in prevalence surveys and equivalence studies were 112 (45–179) and 5.5 (5–8), respectively. Of the 57 data points, there were seven (12.3%) for India, five (8.8%) for Nigeria, four (7.0%) for Saudi Arabia, two (3.5%) each for Jordan and Cambodia, and one (1.8%) each for the other countries (table 2). The country where samples were collected was not specified for 5.5% (n=37) of samples.

**Table 2:** Failure rate per country in prevalence surveys and equivalence studiesBecause of the limited number of samples tested for quality in the studies included in this review, the numbers should not be interpreted as representative of the prevalence of specific SF antidiabetics (please refer to the discussion section of the current paper for more details)

Region/country	Prevalence survey		Equivalence study		Total	
	No of data points	Failure rate % (n/N)	No of data points	Failure rate % (n/N)	No of data points	Failure rate % (n/N)
**Africa**	**2**	**4.2 (8/190)**	**5**	**41.2 (14/34)**	**7**	**9.8 (22/224)**
Egypt	1	9.1 (1/11)	0	N/A	1	9.1 (1/11)
Libya	0	N/A	1	0.0 (0/3)	1	0.0 (0/3)
Nigeria	1	3.9 (7/179)	4	45.2 (14/31)	5	10.0 (21/210)
**Americas**	**4**	**7.5 (3/40)**	**1**	**0.0 (0/4)**	**5**	**6.8 (3/44)**
Argentina	1	37.5 (3/8)	0	N/A	1	37.5 (3/8)
Canada	1	0.0 (0/17)	0	N/A	1	0.0 (0/17)
Chile	1	0.0 (0/11)	0	N/A	1	0.0 (0/11)
Trinidad and Tobago	0	N/A	1	0.0 (0/4)	1	0.0 (0/4)
USA	1	0.0 (0/4)	0	N/A	1	0.0 (0/4)
**Asia and Middle East**	**12**	**12.4 (22/177)**	**12**	**9.2 (7/76)**	**24**	**11.5 (29/253)**
Bangladesh	0	N/A	1	0.0 (0/7)	1	0.0 (0/7)
Cambodia	2	18.8 (21/112)*	0	N/A	2	18.8 (21/112)
CIS	1	0.0 (0/2)	0	N/A	1	0.0 (0/2)
India	4	0.0 (0/45)	3	10.3 (3/29)	7	4.1 (3/74)
Indonesia	1	25.0 (1/4)	0	N/A	1	25.0 (1/4)
Iraq	0	N/A	1	0.0 (0/5)	1	0.0 (0/5)
Japan	1	0.0 (0/4)	0	N/A	1	0.0 (0/4)
Jordan	0	N/A	2	36.4 (4/11)	2	36.4 (4/11)
Malaysia	0	N/A	1	0.0 (0/5)	1	0.0 (0/5)
Pakistan	1	0.0 (0/2)	0	N/A	1	0.0 (0/2)
Saudi Arabia	0	N/A	4	0.0 (0/19)	4	0.0 (0/19)
Thailand	1	0.0 (0/6)	0	N/A	1	0.0 (0/6)
Turkey	1	0.0 (0/2)	0	N/A	1	0.0 (0/2)
**Australia and Oceania**	**1**	**0.0 (0/6)**	**0**	**N/A**	**1**	**0.0 (0/6)**
Australia	1	0.0 (0/6)	0	N/A	1	0.0 (0/6)
**Europe**	**16**	**2.7 (3/110)**	**0**	**N/A**	**16**	**2.7 (3/110)**
Austria	1	0.0 (0/17)	0	N/A	1	0.0 (0/17)
Belgium	1	0.0 (0/2)	0	N/A	1	0.0 (0/2)
Denmark	1	0.0 (0/6)	0	N/A	1	0.0 (0/6)
Finland	1	0.0 (0/5)	0	N/A	1	0.0 (0/5)
France	1	0.0 (0/3)	0	N/A	1	0.0 (0/3)
Germany	1	5.9 (1/17)	0	N/A	1	5.9 (1/17)
Greece	1	0.0 (0/6)	0	N/A	1	0.0 (0/6)
Hungary	1	0.0 (0/2)	0	N/A	1	0.0 (0/2)
Italy	1	0.0 (0/2)	0	N/A	1	0.0 (0/2)
Luxembourg	1	0.0 (0/4)	0	N/A	1	0.0 (0/4)
Netherlands	1	0.0 (0/11)	0	N/A	1	0.0 (0/11)
Portugal	1	0.0 (0/4)	0	N/A	1	0.0 (0/4)
Spain	1	0.0 (0/6)	0	N/A	1	0.0 (0/6)
Sweden	1	0.0 (0/6)	0	N/A	1	0.0 (0/6)
Switzerland	1	0.0 (0/8)	0	N/A	1	0.0 (0/8)
UK	1	18.2 (2/11)	0	N/A	1	18.2 (2/11)
**Unstated**	**1**	**0.0 (0/4)**	**3**	**48.5 (16/33)**	**4**	**43.2 (16/37)**
**Total**	**36**	**6.8 (36/527)**	**21**	**25.2 (37/147)**	**57**	**10.8 (73/674)**

*In Islam 2017,^39^ only the number of medicine failing each quality test was mentioned. Since one medicine may fail more than one test, the failure rate was recorded as the highest possible number of samples failing one of the tests.

CIS, Commonwealth of Independent States; N/A, not applicable; SF, substandard and falsified.

Thirty-six out of 527 (6.8%) and 37 out of 147 (25.2%) samples failed at least one quality test in the prevalence surveys and the equivalence studies, respectively. A total of 73 out of 674 samples (10.8%) analysed were thus SorF. The highest proportion of SorF antidiabetic medicines was observed in Asia and Middle East (11.5%, 29/253), and the lowest proportion was observed in Australia and Oceania with no samples failing, although only one data point was available and six samples were tested for quality. The frequencies of failures in Africa, the Americas and Europe were 9.8% (22/224), 6.8% (3/44) and 2.7% (3/110), respectively. There were 4 out of 57 data points with unknown sampling location.

The largest number of samples collected in prevalence surveys and equivalence studies were of metformin (n=345, 45.5%) and glibenclamide (n=266, 35.1%) (table 3).

**Table 3:** Quality of medicines per API in the included prevalence surveys and equivalence studiesBecause of the limited number of samples tested for quality in the studies included in this review, the numbers should not be interpreted as representative of the prevalence of specific SF antidiabetics (please refer to the discussion section of the current paper for more details)

API	Prevalence survey failure rate (n/N, %)	Equivalence study failure rate (n/N, %)	Total failure rate (n/N, %)
Glimepiride	0/15 (0.0)	13/23 (56.5)	13/38 (34.2)
Sitagliptin	N/A	2/5 (40.0)	2/5 (40.0)
Voglibose	N/A	1/5 (20.0)	1/5 (20.0)
Metformin	14/258 (5.4)	18/89 (20.2)	32/345 (9.3)
Glibenclamide	22/239 (9.2)	3/27 (11.1)	25/266 (9.4)
Gliclazide	0/15 (0.0)	N/A	0/15 (0.0)
**Total**	36/527 (6.8)	37/147 (25.2)	73/674 (10.8)

No studies found for: meglitinide, chlorpropamide, tolbutamide, glipizide, repaglinide, vildagliptin, saxagliptin, pramlintide, empagliflozin, canagliflozin, dapagliflozin, dulaglutide, alogliptin, nateglinide, colesevelam, bromocriptine, albiglutide, lixisenatide, buformin, glibornuride, gliquidone, mitiglinide, miglitol, tolazamide.

API, active pharmaceutical ingredient; N/A, not applicable; SF, substandard and falsified.

Among samples identified in prevalence surveys, the most common reason for failure was API content analysis (n=26/36, 72.2%) (online supplementary file 4). Dissolution, disintegration or drug release failures were the most common failures in equivalence studies (online supplementary file 4), with 54.1% (n=20/37) of samples in equivalence studies failing.

10.1136/bmjgh-2019-001636.supp4 Supplementary data

Out of 40 samples failing API content analysis in the surveys and studies, 16 (40.0%) had low API content and 13 (32.5%) had high API content. The API content was unstated for 11 (27.5%) samples. Twenty-two (55.0%) and 17 (42.5%) samples failing API content analysis were metformin and glibenclamide, respectively. The lowest API content, in comparison to that stated on the packaging, was 82.3% (metformin) and the highest was 111.5% (glibenclamide). Out of 258 samples that had dissolution and/or disintegration and/or drug release testing performed, 27 (10.5%) failed at least one of these tests. Further details on the number of failing samples can be found in online supplementary file 4.

Seven out of 73 samples failing at least one test (9.6%) were substandard, while the rest (n=66, 90.4%) were categorised as SorF medicine since packaging analysis was not performed. In one study, packaging analyses were performed by sending survey findings to manufacturers, but the packaging analyses results pertaining to the authenticity of the medicines were not described.^39^

**Figure 4:**
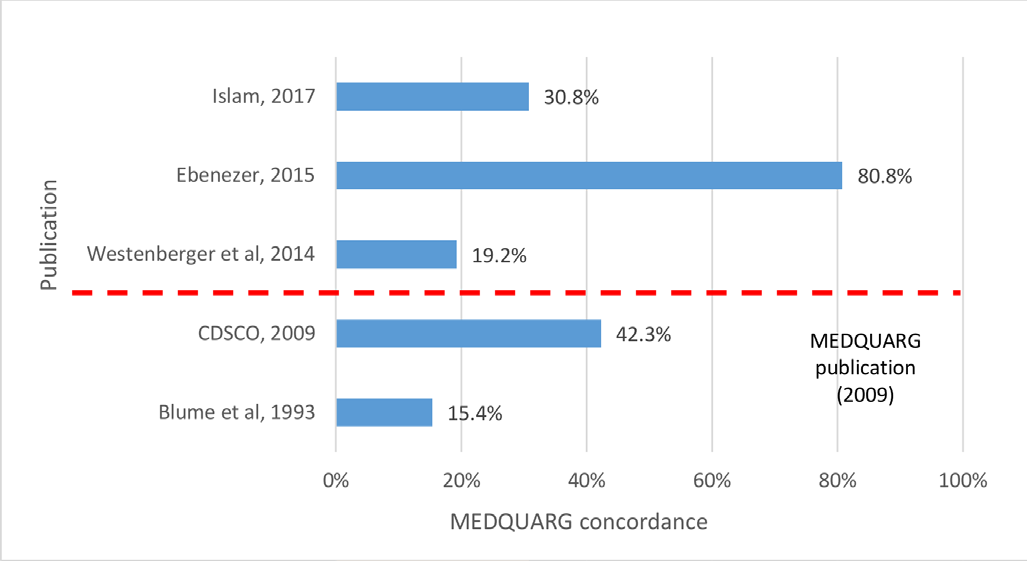
Percentage of concordance of the prevalence surveys with 26 items included in the MEDQUARG checklist. MEDQUARG, Medicine Quality Assessment Reporting Guidelines.

MEDQUARG was mentioned and followed in one of the three (33.3%) prevalence surveys published after the publication of these guidelines in 2009 (figure 4, online supplementary file 5). Only one out of three was published in a scientific journal, with MEDQUARG score of 19.2% and failure rates of 0.0% (0/4 samples).^40^ Four (80.0%) out of five prevalence surveys scored positively on less than half of the MEDQUARG items. The median MEDQUARG score of all surveys was 30.8% (IQR 19.2%–42.3%).

10.1136/bmjgh-2019-001636.supp5 Supplementary data

The time frame, definitions of medicine quality used, ethical considerations, chemical analysis method validation and whether blinding at analysis was performed were described only in one prevalence survey (20.0%) while the sampling methodology and statistical methods were described in two (40.0%). Only two prevalence surveys (40.0%) used random sampling of outlets. Comparative packaging analysis (ie, comparing packaging with that of the authentic medicine from the manufacturer) was performed in three prevalence surveys (60.0%). In one study, label and ‘non-comparative’ packaging analyses were conducted by comparing the samples with the standard US FDA packaging requirements (eg, using label text and package insert requirements).^40^ The outlets types sampled per data point were detailed in four prevalence surveys (80.0%). However, none gave details on the outlet size such as their turnover. The stated manufacturer’s name and address of the collected medicines were given in two prevalence surveys (40.0%), while the rest of the surveys neither give this information nor the reason why the information was not released.

In addition to scientific papers, nine seizures (17.3%), five recalls (9.6%) and two case reports (3.8%) were found (table 4); eight (15.4%) described medicines falsification. Other types of publications discussing antidiabetic medicine quality are presented in online supplementary file 6, including the entries in US Pharmacopeia Medicines Quality Database that lists two reports of the quality of gliclazide from Cambodia in 2008, both samples passed screening tests.^41^

10.1136/bmjgh-2019-001636.supp6 Supplementary data

**Table 4:** Summary of seizures, recalls and case reports of medical products for diabetes management

Study	Report type	Location	API	Quality category and findings
Singh *et al* 107	Case report	South Korea and UK	Insulin (oral)	Substandard: Oral insulin used in clinical trial was adulterated with glibenclamide.
US FDA^108^	Recall	USA	Glibenclamide	Substandard: 45 lots of medicines containing glibenclamide were contaminated by fungus.
SEARPharm Forum Secretariat^109^	Seizure	India	Glibenclamide	Falsified glibenclamide was seized.
SecuringIndustry^20^	Seizure	China	Glibenclamide	Falsified: 9400 bottles seized. High glibenclamide content killed two people.
AboutLawsuits.com^110^	Recall	USA	Metformin	Substandard: 52 lots were contaminated with chemical substances.
Moreno Exebio *et al* 111	Seizure	Peru	Unspecified	Falsified: 4 samples found.
US FDA^112^	Seizure	USA	Metformin/rosiglitazone	Substandard: Coformulated tablets found not to meet the FDA standard formulation mix resulting in higher or lower API content. Manufacturer was also accused of product mix-up, causing tablets with different strength or type to be put in the wrong bottles.
Vanguard^113^	Seizure	Nigeria	Metformin	Falsified: 70 cartons found.
Taylor^114^	Seizure	Multinational	Glimepiride and rosiglitazone, other antidiabetic medicine (unspecified)	Falsified: International operation to address ‘online sales of illicit medicines’ (Operation Pangea). Including inspections and seizures of falsified medicines. Number of antidiabetic medicines not specified.
Woodcock^115^	Case report	USA	Insulin	Suspected degraded: Patient used resold stolen insulin, resulting in ‘poor blood glucose control, likely as a result of it (insulin) not being stored properly’.
Administracion Nacional de Medicamentos, Allmentos y Tecnologia^66^	Recall	Argentina	Insulin	Falsified: 2 lots of insulin; detected through the national medicine tracking system.
Dominican Today^116^	Seizure	Dominican Republic	Unspecified	Falsified: 1 person arrested. ‘Among the drugs seized there are painkillers, antibiotics and others used to combat hypertension and diabetes mellitus.’
Medicines and Healthcare Products Regulatory Agency^117^	Seizure	UK and 114 other countries	Unknown	Falsified: A part of Operation Pangea; 156 arrests made worldwide. ‘Types of medicines seized include […] diabetes […].’
Market^118^	Recall	India	Glimepiride	Substandard: Tablets failed dissolution test.
FDA Philippines^119^	Recall	Philippines	Lixisenatide	Substandard: 3 batches found.
Vanhee *et al* 65	Seizure	Belgium	Insulin	SorF: 20 samples of ‘suspected illegal insulins’ seized by the Belgium Federal Agency for Medicinal and Health Products, porcine insulin with degradation product detected.

API, active pharmaceutical ingredient; FDA, Food and Drug Administration; SorF, substandard or falsified.

We found no prevalence surveys of the quality of SMBG supplies. Of the 29 publications (table 5) on SMBG quality, the majority were recalls/alerts (n=17, 58.6%).^15 42–56^ Sixteen publications described inaccurate blood glucose strip results, resulted in under and overestimation of blood glucose levels, risking wrong treatment doses.^42 48 52 53 55–59^ These quality issues arose through imperfect transportation conditions or sealing, causing glucose strip degradation due to exposure to humidity and/or temperature.^45 51 53^ Contamination by chemical substances during manufacturing resulted in inaccurate results and a recall of 62 million strips.^54^ One manufacturer released an online alert system to monitor falsified products in which poor quality glucose strips with inaccurate/potentially inaccurate test results were reported between 2006 and 2015, in Bangladesh, China, Egypt, Greece, India, United Arab Emirates, Pakistan, France, Singapore and the USA.^60 61^

**Table 5:** The main characteristics of the reviewed publications on the quality of SMBG supplies

Study	Sampling method	Location	Findings
LifeScan, 2006 LifeScan, 2006 Blackwell, 2007 Bloomberg News, 2007 US FDA, 2006^15 43 44 59 120^	Manufacturer investigation (sampling technique unspecified), recall/alert	USA, Canada	Falsified glucose strips imported from China sold in the USA and Canada resulting in incorrect reading. Six lots identified.
Castel and Breillat, 2008^55^	Recall/alert	France	SorF glucose strips: overestimation of blood glucose level, 4 lots affected.
Cheng, 2009^121^	No sampling	USA	Global review on falsified medical products used in diabetes treatment.
Platt, 2009^46^	Recall/alert	USA	Expired and recalled glucose strips were sold by the company that was supposed to recycle them.
LifeScan, 2010^122^	Manufacturer investigation (sampling technique unspecified)	Egypt	Falsified glucose strips giving highly inaccurate results or failing to give result.
Health Sciences Authority, Singapore, 2010^42^	Recall/alert	Singapore	SorF glucose strips: underestimation of blood glucose concentrations.
MacDonald, 2010^56^	Recall/alert	France	SorF glucose strips: underestimation of blood glucose level, 1 lot affected.
Agence Nationale de la Sécurité du Médicament et des Produits de Santé, 2011(a)^53^	Recall/alert	France	SorF glucose strips: resulting in overestimation of blood glucose level, 1 lot affected.
Agence Nationale de la Sécurité du Médicament et des Produits de Santé, 2011(b)^52^	Recall/alert	France	Degraded glucose strips: due to accidental opening of flasks during transport, possible inaccurate result.
Health Sciences Authority, Singapore, 2011^49^	Recall/alert	Singapore	SorF glucose strips: can give inaccurate reading.
Loftus, 2011^47^	Recall/alert	India, Pakistan	Falsified glucose strips manufactured in China were found in India. Falsified strips were also found in 2009.
Mori *et al*, 2011^27^	No sampling	N/A	A review discussing medical device quality in resource-limited settings.
LifeScan, 2013^123^	Manufacturer investigation (sampling technique unspecified)	Greece	Falsified glucose strips: 7 lots found.
NBC News, 2013^54^	Recall/alert	USA	Substandard glucose strips: chemical contamination of strips distributed to 13 countries.
US FDA, 2013^57^	Case report	USA	SorF glucose meter and strips: overestimation of blood glucose concentrations
US FDA, 2013^48 58^	Recall/alert	USA	Twenty-one lots of SorF glucose strips were found. When used, the strips showed incorrectly low readings.
LifeScan, 2015^124^	Manufacturer investigation (sampling technique unspecified)	Bangladesh	Falsified glucose strips: 5 lots found.
LifeScan, 2015^125^	Manufacturer investigation (sampling technique unspecified)	India	Falsified glucose strips: possible falsification within 4 lot numbers.
FDA News, 2016^45^	Recall/alert	UK	Improperly sealed glucose strips found circulating.
Nipro Asia Pte Ltd, 2016^51^	Recall/alert	Singapore	Degraded glucose strips: may give inaccurate result. Degraded due to opened vials during transport, 6 lots affected.
PTS Diagnostics, 2016^50^	Recall/alert	Singapore	SorF glucose strips: giving inaccurate reading
LifeScan, unknown^60^	Manufacturer investigation (sampling technique unspecified)	Bangladesh, China, Egypt, Greece, India and UAE	Describing falsified LifeScan glucose strips.
LifeScan, unknown^61^	No sampling	N/A	Manufacturer policies to fight poor quality medicines through four processes, namely: distribution, identification, enforcement, prevention.
SafeMedicines, unknown^126^	No sampling	USA, Canada, India, Egypt, Pakistan	Articles discussing falsified medical products used in the treatment of diabetes (including glucose strips) found globally.

FDA, Food and Drug Administration; N/A, not applicable; SMBG, self-monitoring of blood glucose; SorF, substandard or falsified; UAE, United Arab Emirates.

## Discussion

We synthesised, from diverse publicly accessible sources, the available evidence on the quality of antidiabetic medicines and SMBGs. The data available are very meagre and do not allow an accurate synthesis of the current situation. However, these findings raise concern and suggest that more evidence is needed to inform policy and interventions. Around 1/10 of the antidiabetic medicines analysed in 5 prevalence surveys and 20 equivalence studies failed analyses. Quality issues related to SMBG supplies included falsified products and incorrect results due to strip degradation or contamination. With the large and increasing global burden of diabetes and the increasing use of antidiabetics globally, the published evidence clearly shows the lack of available evidence to assess the extent of the problem and to identify actions towards ensuring good quality antidiabetics and SMBG. Efforts to increase access to antidiabetic medicines and consumables, especially insulin in LMICs,^5^ need to be linked to ensuring their quality.

We found no prevalence surveys on SMBG supply quality and only five publicly available surveys aiming to assess the quality of antidiabetics available in the market. Only three published since 2010, among which only one was published in scientific journals.^40^ The total number of antidiabetic medicine samples analysed in the five included surveys was very small (674 samples) in comparison to the vast use of antidiabetic medicines worldwide, estimated to be worth >US$50 billion in 2017.^62 63^ This number fell down to four if considering only the single survey published in scientific journals after the publication of MEDQUARG, with none of the four samples failing any tests.

For insulin alone, the demand in 2014 was 2.15 billion vials.^6^ However, we retrieved only one publicly available report on insulin quality. This report, published in 1979, showed that the 64 ‘conventional insulin preparations’ tested contained ‘considerable amounts’ of several hormonal peptide contaminants including pancreatic glucagon, pancreatic polypeptide, vasoactive intestinal peptide and somatostatin.^38^ In the past, insulins were extracted and purified from animals. These contaminants are now avoided through newer manufacturing methods, for example, recombinant human insulin.^64^ This report reminds us that medicines deemed standard today may be deemed substandard in the future, as the standards applicable to medicines evolve. As there were uncertainties regarding the reference acceptance ranges for the amount of contaminants found, this study was excluded from our analysis.

The recent description of suspected illegal insulin seized by inspectors in Belgium is of concern.^65^ The quality of insulin delivery devices such as insulin pens and pen needles is outside of the scope of this review. However, the finding of falsified insulin pens in Argentina, and insulin pen needles in the UK, Poland and the Netherlands are worrying–highlighting the need for postmarketing surveillance of the quality of these products.^66–69^

We only included five prevalence surveys, in which glibenclamide, metformin, gliclazide and glimepiride were assessed, with none for numerous other antidiabetic medicines (see table 3). With a median of 112 samples collected in the five prevalence surveys analysed in this review—only two using random sampling—a global estimate of the prevalence of poor quality antidiabetics is thus not possible. However, since there should be no poor quality medicines or medical products circulating, the finding of any poor quality samples indicates a problem that needs further investigation.

There were only four data points from the Americas, despite North America and the Caribbean having the highest age adjusted adult prevalence of diabetes.^1^ Seven countries with high adult diabetes prevalence (20% or more) have no data points (ie, Kuwait, Qatar, Cook Islands, Marshall Islands, Nauru, Palau and Tokelau).^1^ China has the largest number of patients with diabetes globally^1^. No published prevalence survey of the quality of antidiabetic medications in the Chinese pharmaceutical supply chain was identified, although lethal cases of falsified glibenclamide, containing dangerously high glibenclamide content, were reported in 2009.^20 21^

The quality of reporting was poor, as reflected by the low MEDQUARG scores. Survey design and results were not well described, making it difficult to judge the evidence. Random sampling, that will minimise bias, was used in only two of the prevalence surveys reviewed, probably because random surveys require more resources compared with convenience surveys. A more economical but objective approach would be to use lot quality assurance sampling (LQAS), as a ‘screening’ step, to determine if the prevalence of poor quality medicines exceeds a predefined threshold, but LQAS cannot give an accurate estimate of prevalence.^36^

Only one of the surveys clearly delineate the classification of samples as SF. In most of the surveys and all the equivalence studies, quality was determined only by chemical analysis, without packaging analysis which is vital for distinguishing falsified and substandard medicines, essential data for determining actions.

Limitations of this review include that searches were conducted only in English and French, and that medicine regulatory authorities and the pharmaceutical industry are likely to have significant amounts of data not shared with the public. There may be unpublished data if no SF antidiabetics were identified in a survey, or if SF antidiabetics were found but public release may have been considered damaging to the country or pharmaceutical company.^22^ The publicly available data on antidiabetics quality included in this review are scarce, of low quality, and the studies varied greatly in methodology. Only two surveys were conducted using random sampling. Therefore, the summary presented in this work should be considered with caution. One of the variation is the reference standard used. In one study, manufacturer specifications of the innovator’s product were used as a reference to assess the quality of generic products. However, if acceptance ranges of the USP monograph from 2017 had been followed, the number of samples failing dissolution test would be reduced from 12 out of 23 (52.2%) to 8 out of 23 (34.8%).^70^ In most prevalence surveys, we found limited detailed information on samples and/or samples quality with breakdowns by outlet sampled (eg, licensed vs unregistered outlet), cost of medicines or country of manufacture. We thus did not perform causal factors analysis that, although crucial to better inform policy, could lead to misleading results and interpretation.

Poor quality antidiabetic medicines containing lower amounts of API than stated, or poor dissolution rate engendering reduced bioavailability will, as for non-adherent patients, risk developing macrovascular and microvascular complications,^17 71^ and severe infections, such as pneumonia, tuberculosis or melioidosis.^17 72 73^ Complication-related hospitalisations will further increase costs to patients and society.^74^

The USP 40 and the Chinese Pharmacopoeia (2010) percentage API acceptance range is 95%–105% for metformin and 90%–110% for glibenclamide tablets.^75 76^ There is limited evidence for the dose–response relationship for either medicine,^77–79^ making it hard to predict the relative risk of acute or regular underdosing and overdosing for either medicine. However, underdosing is likely to impair glycaemic control and with the inherent risk of hypoglycaemia with most of the oral antidiabetic medicines (especially with sulfonylureas such as glibenclamide) at normal doses, those containing high percentage API are especially dangerous.^80^ Glibenclamide has also been found in falsified erectile dysfunction drugs with devastating lethal hypoglycaemic consequences.^81^ Unexplained hypoglycaemia with new medicines, brand or batch numbers should prompt investigation of their contents.

Higher failure rates were found in previous reviews.^22 23 82 83^ Almost one-third of antimalarials tested failed either packaging or chemical tests in a review published in 2014.^22^ Failure rates of 19.1% and 12.4% were observed in a review of 96 articles for antimalarials and antibiotics, respectively.^83^ Although the number of publications gathered for these reviews were much larger than for our review, authors also concluded that the estimate of the prevalence and distribution of SF medicines could not be accurate because of the heterogeneity of study methodology, as illustrated by an I-square parameter of 0.99 in a recent meta-analysis.^83^

The evaluation of glucose meters and strips quality is complex since glucose readings are influenced by multiple factors, including the analytical properties of the device, the calibration and setting of the device and user compliance with testing procedures.^84^ The currently available standards for glucose meter and strip performance and scientific literature mainly focus on the accuracy of the device, overlooking user error or the clinical impact of inaccuracies.^84^ As with medicines, consensus on a standardised definition and methods to evaluate the quality of SMBG supplies and other medical devices must be established to enable a more valid prevalence estimation.^27 30^

Another notable issue is the unauthorised reselling of unused glucose strips. The advocates of this practice argue that this facilitates strip access for the poorest, as resold glucose strips are sold for less than the standard market price.^10^ However, it has been argued that unauthorised reselling risks improper storage, exposing strips to humidity and heat potentially affecting accuracy. One company strategy of proactively detecting circulating poor quality SMBG supplies and coordinating with governmental agencies to enforce regulation^61^ has resulted in detection of falsified products.^60^ Such models of surveillance and coordination are needed to effectively tackle the problem of SF medicines and medical products.

## Conclusion

The currently available data on the quality of antidiabetic medicines and consumables are sparse and of variable quality, suggesting that aggregated data should be interpreted cautiously. However, despite the relatively small number of publicly available studies, poor quality antidiabetic medicines and SMBG supplies were identified on four continents, suggesting that this is an important public health issue and should be further investigated, through factory inspections and postmarket surveillance, to ensure that the benefits of modern diabetes management are fulfilled for the burgeoning global population with diabetes.

## References

[1] Cavan D, da Rocha Fernandes J, Makaroff L, et al IDF diabetes atlas. 7th ed Brussels: International Diabetes Federation, 2015 http://www.diabetesatlas.org/resources/2015-atlas.html

[2] World Health Organization Global report on diabetes. Available: http://apps.who.int/iris/bitstream/handle/10665/204871/9789241565257_eng.pdf?sequence=1 [Accessed 25 Nov 2018].

[3] Center of Disease Control Age-Adjusted percentage of adults with diabetes using diabetes medication, by type of medication, United States, 1997-2011. Available: http://www.cdc.gov/diabetes/statistics/meduse/fig2.htm [Accessed 9 Aug 2016].

[4] Kollewe J The world’s 10 best-selling prescription drugs. Available: https://www.theguardian.com/business/table/2014/mar/27/world-best-selling-prescription-drugs-pharmaceuticals-industry [Accessed 20 Jul 2016].

[5] Beran D, Ewen M, Laing R Constraints and challenges in access to insulin: a global perspective. Lancet Diabetes Endocrinol 2016;4:275–85. 10.1016/S2213-8587(15)00521-5 26857998

[6] Wirtz VJ, Knox R, Cao C, et al Insulin market profile. Amsterdam: health action international, 2016 http://haiweb.org/wp-content/uploads/2016/04/ACCISS_Insulin-Market-Profile_FINAL.pdf [Accessed 12 Feb 2017].

[7] Dayan C, Williams G Diabetes : Warrell D, Cox T, Firth J, Oxford textbook of medicine. 5th ed Oxford: Oxford University Press, 2010.

[8] GBD 2013 Mortality and Causes of Death Collaborators Global, regional, and national age-sex specific all-cause and cause-specific mortality for 240 causes of death, 1990-2013: a systematic analysis for the global burden of disease study 2013. Lancet 2015;385:117–71. 10.1016/S0140-6736(14)61682-2 25530442 PMC4340604

[9] Zion Market Research Global oral antidiabetic drug market is set for a rapid growth and is expected to reach USD 35.91 billion by 2022. Available: https://www.zionmarketresearch.com/news/oral-anti-diabetic-drug-market [Accessed 27 Jan 2018].

[10] Cheng MM, Gedeon C Counterfeit diabetes products and the ethical question of access. Diabetes Manag 2015;5:341–7. 10.2217/dmt.15.24

[11] Bate R Phake: the deadly world of falsified and substandard medicines. Washington D.C: The AEI Press, 2012.

[12] NCD Risk Factor Collaboration (NCD-RisC) Worldwide trends in diabetes since 1980: a pooled analysis of 751 population-based studies with 4.4 million participants. Lancet 2016;387:1513–30. 10.1016/S0140-6736(16)00618-8 27061677 PMC5081106

[13] Yeaw J, Lee WC, Aagren M, et al Cost of self-monitoring of blood glucose in the United States among patients on an insulin regimen for diabetes. J Manag Care Pharm 2012;18:21–32. 10.18553/jmcp.2012.18.1.21 22235952 PMC10438111

[14] Walker A Resold diabetes strips cause health concerns. Available: http://articles.baltimoresun.com/2014?05?31/news/bs?hs?diabetes?test?strips?20140531_1_test?strips?the?fda?radiological?health [Accessed 12 Apr 2017].

[15] Blackwell T Firm suing over fake diabetes test strips. Available: http://www.canada.com/nationalpost/news/story.html?id=2edf7f4c-2b09-4a4d-bd85-8f07235e0ca6&k=37047 [Accessed 18 Sep 2007].

[16] US FDA How to safely use glucose meters and test strips for diabetes

[17] Stratton IM, Adler AI, Neil HA, et al Association of glycaemia with macrovascular and microvascular complications of type 2 diabetes (UKPDS 35): prospective observational study. BMJ 2000;321:405–12. 10.1136/bmj.321.7258.405 10938048 PMC27454

[18] Intensive blood-glucose control with sulphonylureas or insulin compared with conventional treatment and risk of complications in patients with type 2 diabetes (UKPDS 33). The Lancet 1998;352:837–53. 10.1016/S0140-6736(98)07019-6 9742976

[19] Leal J, Luengo-Fernández R, Gray A, et al Economic burden of cardiovascular diseases in the enlarged European Union. Eur Heart J 2006;27:1610–9. 10.1093/eurheartj/ehi733 16495286

[20] SecuringIndustry Fake diabetes drug kills two in China. Available: https://www.securingindustry.com/fake-diabetes-drug-kills-two-in-china/s15/a81/ [Accessed 12 Jun 2016].

[21] Xinhua News Agency Fake diabetes drug hospitalizes 9 in Xinjiang. Available: http://china.org.cn/china/news/2009-02/04/content_17220020.htm [Accessed 19 Feb 2016].

[22] Tabernero P, Fernández FM, Green M, et al Mind the gaps--the epidemiology of poor-quality anti-malarials in the malarious world--analysis of the WorldWide Antimalarial Resistance Network database. Malar J 2014;13:139 10.1186/1475-2875-13-139 24712972 PMC4021408

[23] Newton PN, Green MD, Fernández FM, et al Counterfeit anti-infective drugs. Lancet Infect Dis 2006;6:602–13. 10.1016/S1473-3099(06)70581-3 16931411

[24] Cockburn R, Newton PN, Agyarko EK, et al The global threat of counterfeit drugs: why industry and governments must communicate the dangers. PLoS Med 2005;2:e100–8. 10.1371/journal.pmed.0020100 15755195 PMC1062889

[25] Attaran A, Barry D, Basheer S, et al How to achieve international action on falsified and substandard medicines. BMJ 2012;345:e7381 10.1136/bmj.e7381 23149211

[26] Nayyar GML, Attaran A, Clark JP, et al Responding to the pandemic of falsified medicines. Am J Trop Med Hyg 2015;92(6 Suppl):113–8. 10.4269/ajtmh.14-0393 25897060 PMC4455086

[27] Mori M, Ravinetto R, Jacobs J Quality of medical devices and in vitro diagnostics in resource-limited settings. Trop Med Int Health 2011;16:1439–49. 10.1111/j.1365-3156.2011.02852.x 21955331

[28] WHO WHO global surveillance and monitoring system for substandard and falsified medical products. Geneva World Health Organization; 2017.

[29] McGinnis M Media reports on medicine quality: Focusing on USAID-assisted countries by the promoting the quality of medicines program

[30] Newton PN, Amin AA, Bird C, et al The Primacy of public health considerations in defining poor quality medicines. PLoS Med 2011;8:e1001139 10.1371/journal.pmed.1001139 22162953 PMC3232210

[31] Fahad A, Alghannam A, Aslanpour Z, et al A systematic review of counterfeit and substandard medicines in field quality surveys. Integr Pharm Res Pract 2014;3:71–88.

[32] Antignac M, Diop BI, Macquart de Terline D, et al Fighting fake medicines: first quality evaluation of cardiac drugs in Africa. Int J Cardiol 2017;243:523–8. 10.1016/j.ijcard.2017.04.099 28641892

[33] Gyanwali P, Humagain BR, Aryal KK, et al Surveillance of quality of medicines available in the Nepalese market: a study from Kathmandu Valley. J Nepal Health Res Counc 2015;13:233–40. 27005718

[34] World Health Organization WHO member state mechanism on substandard/spurious/falseabelled/falsified/counterfeit (SSFFC) medical products. Available: https://www.who.int/medicines/regulation/ssffc/A70_23-en1.pdf?ua=1 [Accessed 25 Nov 2018].

[35] World Health Organization Report number: EB124/14 Counterfeit medical products World Health Organization; 2008.

[36] Newton PN, Lee SJ, Goodman C, et al Guidelines for field surveys of the quality of medicines: a proposal. PLoS Med 2009;6:e1000052–257. 10.1371/journal.pmed.1000052 PMC2659710 19320538

[37] Moher D, Liberati A, Tetzlaff J, et al Preferred reporting items for systematic reviews and meta-analyses: the PRISMA statement. PLoS Med 2009;6:e1000097 10.1371/journal.pmed.1000097 19621072 PMC2707599

[38] Bloom SR, Adrian TE, Barnes AJ, et al Autoimmunity in diabetics induced by hormonal contaminants of insulin. Lancet 1979;1:14–17. 10.1016/s0140-6736(79)90455-0 83463

[39] Islam MR Investigations of the quality medicines distributed in Myanmar and Cambodia, through different surveys [PhD thesis]. Kanazawa University Repository for Academic Resources, 2017.

[40] Westenberger BJ, Ellison CD, Fussner AS, et al Quality assessment of Internet pharmaceutical products using traditional and non-traditional analytical techniques. Int J Pharm 2005;306(1-2):56–70. 10.1016/j.ijpharm.2005.08.027 16266793

[41] United States Pharmacopeial Convention Medicines quality database. Available: http://apps.usp.org/app/worldwide/medQualityDatabase/reportResults.html?country=Ethiopia%2BGhana%2BKenya%2BMozambique%2BNigeria%2BCambodia%2BLao+PDR%2BPhilippines%2BThailand%2BViet+Nam%2BBolivia%2BColombia%2BEcuador%2BGuatemala%2BGuyana%2BPeru&period=2017 [Accessed 17 Dec 2018].

[42] Health Sciences Authority HSA alerts public on Abbott’s voluntary recall of Medisense OptiumTM blood glucose test strips. Available: http://www.hsa.gov.sg/content/dam/HSA/News_and_Events/Press_Releases/2010/PressRelease-HSAAlertsPublicOnAbbottsVoluntaryRecallOfMedisenseOptiumBloodGlucoseTestStrips_24Dec10.pdf [Accessed 19 Apr 2017].

[43] The New York Times Bogus diabetes test strips traced to Chinese distributor. Available: http://www.nytimes.com/2007/08/17/business/worldbusiness/17fraud.html?_r=0[Accessed 19 Feb 2016].

[44] FDA Class 1 device recall one touch. Available: https://www.accessdata.fda.gov/scripts/cdrh/cfdocs/cfRES/res.cfm?id=49472 [Accessed 26 Sep 2017].

[45] FDA News UK warns on Nipro glucose strips. Available: http://www.fdanews.com/articles/178060-uk-warns-on-nipro-glucose-strips [Accessed 26 Sep 2017].

[46] Platt S Knox CEO pleads guilty to fraud, illegally selling diabetic testing strips. Available: http://www.wndu.com/home/headlines/37185719.html [Accessed 29 Sep 2017].

[47] Loftus J&J finds fake diabetes test strips. Available: https://www.wsj.com/articles/SB10001424052748703778104576286921573327708#articleTabs%3Darticle [Accessed 26 Sep 2017].

[48] FDA Class 1 device recall FreeStyle blood glucose test strips. Available: https://www.accessdata.fda.gov/scripts/cdrh/cfdocs/cfRES/res.cfm?id=123972 [Accessed 26 Sep 2017].

[49] Health Sciences Authority HSA alerts public on Bayer’s voluntary recall of Bayer ContourTMTS 2x25-count, 25-count, 10-count test strip vials. Available: http://www.hsa.gov.sg/content/dam/HSA/News_and_Events/HSA_Updates/2011/HSAUpdates_HSAAlertsPublicOnBayerVoluntaryRecallOfBayerContourTestStripVials-24Aug2011.pdf [Accessed 19 Apr 2017].

[50] PTS Diagnostics Field corrective action 16-001, customer notification letter. Available: http://www.hsa.gov.sg/content/dam/HSA/HPRG/Medical_Devices/Updates_and_Safety_reporting/Field_Safety_Corrective_Action/FSN/2016/May/HSA 6004101-186-16-01_58 FSN.pdf[Accessed 19 Apr 2017].

[51] Nipro Asia Pte Ltd Field safety notice. Available: http://www.hsa.gov.sg/content/dam/HSA/HPRG/Medical_Devices/Updates_and_Safety_reporting/Field_Safety_Corrective_Action/FSN/2016/May/HSA 6004101-233-16-03_31 FSN.pdf [Accessed 2 Feb 2017].

[52] Agence Nationale de la Sécurité du Médicament et des Produits de Santé Rappel d’un lot de bandelettes Accu-Chek® Performa (Roche Diagnostics France) pour lecteurs de glycémie. Available: http://www.ansm.sante.fr/S-informer/Presse-Communiques-Points-presse/Rappel-d-un-lot-de-bandelettes-Accu-Chek-R-Performa-Roche-Diagnostics-France-pour-lecteurs-de-glycemie-Communique [Accessed 19 April 2017].

[53] Agence Nationale de la Sécurité du Médicament et des Produits de Santé Rappel de plusieurs lots de bandelettes mylifeTM Pura® Boites de 100 pour lecteurs de glycémie mylifeTMPura®. Available: http://ansm.sante.fr/var/ansm_site/storage/original/application/cbc882b85b6e0b812a1a992f47639b79.pdf [Accessed 19 April 2017].

[54] NBC News FDA warns of massive diabetes test strip recall. Available: http://www.nbcnews.com/health/diabetes/fda-warns-massive-diabetes-test-strip-recall-f6C10812167 [Accessed 19 Apr 2017].

[55] Castel I, Breillat P Rappel des bandelettes Du lecteur de glycémie contour Ts. Available: http://ansm.sante.fr/var/ansm_site/storage/original/application/13e131c860c3f4887cc896762defda5d.pdf [Accessed 19 Apr 2017].

[56] Agence Nationale de la Sécurité du Médicament et des Produits de Santé Retrait d’un lot de bandelettes pour lecteur de glycémie Nova StatStrip Glucose Test Strips de la société Nova Biomédical. Available: http://ansm.sante.fr/S-informer/Informations-de-securite-Retraits-de-lots-et-de-produits/Retrait-d-un-lot-de-bandelettes-pour-lecteur-de-glycemie-Nova-StatStrip-Glucose-Test-Strips-de-la-societe-Nova-Biomedical [Accessed 19 Apr 2017].

[57] FDA Prodigy diabetes care, LLC 2/22/13. Available: https://www.fda.gov/ICECI/EnforcementActions/WarningLetters/2013/ucm360148.htm [Accessed 19 Apr 2017].

[58] FDA Class 1 device recall FreeStyle Lite blood glucose test strips. Available: https://www.accessdata.fda.gov/scripts/cdrh/cfdocs/cfRES/res.cfm?id=123985 [Accessed 26 Sep 2017].

[59] LifeScan Counterfeit test strips discovered in United States: OneTouch® Ultra® and OneTouch® Basic®/Profile® test strips. Available: https://www.lifescan.com/about-us/news/149 [Accessed 26 Sep 2017].

[60] LifeScan Counterfeit OneTouch® test strip alerts. Available: http://www.lifescan.com/responsibility/counterfeits/alerts [Accessed 16 Apr 2017].

[61] LifeScan How LifeScan is fighting counterfeit products. Available: http://www.lifescan.com/responsibility/counterfeits/how-lifescan [Accessed 19 Apr 2017].

[62] Visiongain Diabetes drugs market will reach $55.3bn in 2017, with further growth to 2023, predicts Visiongain in new report

[63] Grand View Research Antidiabetics market analysis by product (insulin, biguanides, thiazolodinediones, GLP-agonists, sulphonylureas, DPP-4 inhibitors, SGLT-2, alpha-glucosidase inhibitors, meglitinides) and segment forecasts to 2020. Available: http://www.grandviewresearch.com/industry?analysis/antidiabetics?market [Accessed 12 Apr 2017].

[64] Baeshen NA, Baeshen MN, Sheikh A, et al Cell factories for insulin production. Microb Cell Fact 2014;13:141 10.1186/s12934-014-0141-0 25270715 PMC4203937

[65] Vanhee C, Janvier S, Moens G, et al A simple dilute and shoot methodology for the identification and quantification of illegal insulin. J Pharm Anal 2016;6:326–34. 10.1016/j.jpha.2016.04.006 29404000 PMC5762622

[66] Administracion Nacional de Medicamentos Allmentos y Tecnologia Medica Anmat advierte sobre unidades ilegítimas de insulina lantus. Available: http://www.anmat.gov.ar/comunicados/lantus_insulina.pdf [Accessed 16 Jun 2016].

[67] Hirschler B Counterfeit novo Nordisk insulin pens discovered. Available: https://uk.reuters.com/article/uk-britain-counterfeit/counterfeit-novo-nordisk-insulin-pens-discovered-idUKTRE52Q3LZ20090327

[68] The Pharmaceutical Journal Medical device alert: counterfeit insulin pen needle warning. Available: https://www.pharmaceutical-journal.com/news-and-analysis/news/medical-device-alert-counterfeit-insulin-pen-needle-warning/10794479.article [Accessed 29 Apr 2018].

[69] SafeMedicines Warning for counterfeit insulin Pens. Available: https://www.safemedicines.org/2009/07/warning-for-counterfeit-insulin-pens-.html [Accessed 29 April 2018].

[70] Attorrese G, Massi-Benedetti M Quality and behavior of glimepiride generics versus amaryl under stressed conditions. Diabetes Technol Ther 2007;9:287–96. 10.1089/dia.2006.0029 17561799

[71] Fukuda H, Mizobe M Impact of nonadherence on complication risks and healthcare costs in patients newly-diagnosed with diabetes. Diabetes Res Clin Pract 2017;123:55–62. 10.1016/j.diabres.2016.11.007 27940390

[72] van Crevel R, van de Vijver S, Moore DAJ The global diabetes epidemic: what does it mean for infectious diseases in tropical countries? Lancet Diabetes Endocrinol 2017;5:457–68. 10.1016/S2213-8587(16)30081-X 27499355 PMC7104099

[73] Dronge AS, Perkal MF, Kancir S, et al Long-Term glycemic control and postoperative infectious complications. Arch Surg 2006;141:375–80. 10.1001/archsurg.141.4.375 16618895

[74] García-Pérez L-E, Álvarez M, Dilla T, et al Adherence to therapies in patients with type 2 diabetes. Diabetes Ther 2013;4:175–94. 10.1007/s13300-013-0034-y 23990497 PMC3889324

[75] United States Pharmacopeial Convention United States pharmacopeia and national formulary (USP 40-NF 35), 2017, 2016 Available: http://www.uspnf.com [Accessed 29 Dec 2018].

[76] (2010). Pharmacopeia of the People’s Republic of China.

[77] Scarpello JH Review: optimal dosing strategies for maximising the clinical response to metformin in type 2 diabetes. Br J Diabetes Vasc Dis 2001;1:28–36. 10.1177/14746514010010010501

[78] Groop LC Sulfonylureas in NIDDM. Diabetes Care 1992;15:737–54. 10.2337/diacare.15.6.737 1600834

[79] Garber AJ, Duncan TG, Goodman AM, et al Efficacy of metformin in type II diabetes: results of a double-blind, placebo-controlled, dose-response trial. Am J Med 1997;103:491–7. 10.1016/s0002-9343(97)00254-4 9428832

[80] Holstein A, Egberts E-H Risk of hypoglycaemia with oral antidiabetic agents in patients with type 2 diabetes. Exp Clin Endocrinol Diabetes 2003;111:405–14. 10.1055/s-2003-44287 14614647

[81] Kao SL, Chan CL, Tan B, et al An unusual outbreak of hypoglycemia. N Engl J Med 2009;360:734–6. 10.1056/NEJMc0807678 19213693

[82] Kelesidis T, Falagas ME Substandard/counterfeit antimicrobial drugs. Clin Microbiol Rev 2015;28:443–64. 10.1128/CMR.00072-14 25788516 PMC4402958

[83] Ozawa S, Evans DR, Bessias S, et al Prevalence and estimated economic burden of substandard and falsified medicines in low- and middle-income countries: a systematic review and meta-analysis. JAMA Netw Open 2018;1:e181662 10.1001/jamanetworkopen.2018.1662 30646106 PMC6324280

[84] Krouwer JS, Cembrowski GS A review of standards and statistics used to describe blood glucose monitor performance. J Diabetes Sci Technol 2010;4:75–83. 10.1177/193229681000400110 20167170 PMC2825627

[85] Blume H, Ali SL, Siewert M Pharmaceutical quality of glibenclamide products a multinational Postmarket comparative study. Drug Dev Ind Pharm 1993;19:2713–41. 10.3109/03639049309050174

[86] Central Drugs Standard Control Organisation Report on countrywide survey for spurious drugs. Central Drugs Standard Control Organisation

[87] Ebenezer CJ Pharmaceutical quality and policy in Nigeria: Stakehoder perspective and validation of the mobile authentication service [PhD thesis]. London, University College London, 2015.

[88] Hamdan II, Jaber AKB Pharmaceutical evaluation of metformin HCl products available in the Jordanian market. Jordan J Pharm Sci 2010;3:1–7.

[89] Chandrasekaran AR, Jia CY, Theng CS, et al Invitro studies and evaluation of metformin marketed tablets-Malaysia. J Appl Pharm Sci 2011;1:214–7.

[90] Afifi SA, Ahmadeen S A comparative study for evaluation of different brands of metformin hydrochloride 500 Mg tablets marketed in Saudi Arabia. Life Sci J 2012;9:4260–6.

[91] Chatur VM, Ghodekar S, Kadam P, et al Quality evaluation of marketed formulation of mouth dissolving voglibose tablets. IJPBCS 2012;1:1–4.

[92] Olusola AM, Adekoya AI, Olanrewaju OJ Comparative evaluation of physicochemical properties of some commercially available brands of metformin HCl tablets in Lagos, Nigeria. J Appl Pharm Sci 2012;2:41–4.

[93] Oyetunde OO, Tayo F, Akinleye MO, et al In vitro equivalence studies of generic metformin hydrochloride tablets and propranolol hydrochloride tablets under Biowaiver conditions in Lagos state, Nigeria. Dissolut Technol 2012;19:51–5. 10.14227/DT190412P51

[94] El-Sabawi D, Abbasi S, Alja’fari S, et al Pharmaceutical evaluation of metformin HCl products available in the Jordanian market. Jordan J Pharm Sci 2010;3:1–7.

[95] Labu ZK, Debnath P, Basir MS, et al Analytical appraisement of some commercially available brands of metformin hydrochloride tablet in Dhaka, Bangladesh. Asian J Chem Pharm Res 2013;1:47–52.

[96] Ajala TO, Adebona AC, Bamiro OA The pharmaceutical quality of brands of metformin tablets in Ogun-State, Nigeria. Afr J Biomed Res 2014;17:43–8.

[97] Betari N, Haidar S Pharmaceutical quality of generic sitagliptin tablets compared with Januvia. J App Pharm 2014;6:195–201. 10.21065/19204159.6.3.172

[98] Elango P, Shanmuganathan S A comparative analysis of commercial metformin tablets. Indian Journal of Clinical Practice 2014;24:778–83.

[99] Elhamili A, Bergquist J, Saad S, et al Pharmaceutical evaluation of type II oral antidiabetic agent. IJPRR 2014;3:1–9.

[100] Abdulhameed BR, Karim HI, Qader GI, et al In vitro equivalence evaluation of five different metformin tablet products using UV spectrometer. JSMC 2016;6:155–61. 10.17656/jsmc.10100

[101] Gupta MM, Gupta M In-vitro pharmaceutical quality control testing: A comparative study of different brands of metformin tablets available in the Trinidad & Tobago, West Indies. J Pharm Sci Res 2016;8:238–43.

[102] Sachan AK, Kumar V, Gupta A Comparative in-vitro evaluation of four different brands of metformin HCl available in Kanpur district, India. Pharm Lett 2016;8:419–24.

[103] Sakr F, Alobaidy K, Almarri A, et al Evaluation of the quality and pharmacoeconomics of some generic drugs versus their reputed counterpart brands in the Saudi market. Saudi J Oral Sci 2016;3:97–103. 10.4103/1658-6816.188081

[104] Alam MN, El-Gied AAA, Rahamathulla M, et al A comparative in vitro dissolution of different brands of glibenclamide tablets available in Saudi Arabian market. Int J Adv Pharm Med Bioallied Sci 2017;2017:1–5.

[105] Eraga SO, Arhewoh MI, Oruh EP, et al A comparative evaluation of the pharmaceutical quality of different brands of metformin hydrochloride tablets available in Abuja, Nigeria. West African J Pharm 2017;28:61–71.

[106] Aivalli PK, Elias MA, Pati MK, et al Perceptions of the quality of generic medicines: implications for trust in public services within the local health system in Tumkur, India. BMJ Glob Health 2018;2(Suppl 3):e000644 10.1136/bmjgh-2017-000644 PMC5844374 29531844

[107] Singh BM, Wise PH, Marks V Detection of glibenclamide in oral insulin capsules. Lancet 1991;338:308–10. 10.1016/0140-6736(91)90448-X 1677127

[108] Center for Drug Evaluation and Research Inspections, compliance, enforcement, and criminal investigations. Available: http://www.fda.gov/ICECI/EnforcementActions/EnforcementStory/EnforcementStoryArchive/ucm107053.htm [Accessed 12 Sep 2016].

[109] SEARPharm Forum Secretariat Database on the incidents of counterfeit medicines in the WHO-SEA region; 2004.

[110] AboutLawsuits.com Glumetza recall: 52 lots of diabetes drug may have chemical contamination. Available: http://www.aboutlawsuits.com/glumetzarecallcontamination10917/[Accessed 16 Jun 2016].

[111] Moreno Exebio LE, Rodríguez J, Sayritupac F Los medicamentos falsificados en Perú. Rev Panam Salud Pública 2010;27:138–13. 20339618 10.1590/s1020-49892010000200008

[112] FDA GlaxoSmithKline will plead guilty and pay $750 Million to resolve manufacturing deficiencies at Puerto Rico plant. Available: https://www.fda.gov/ICECI/CriminalInvestigations/ucm231523.htm [Accessed 16 Jun 2017].

[113] Vanguard NAFDAC uncovers illegal drug factory in Onitsha. Available: http://www.vanguardngr.com/2012/05/nafdac?uncovers?illegal?drug?factory?in?onitsha/ [Accessed 16 Jun 2016].

[114] Taylor P Pangea VI swoop nets $ 41m-worth of fake medicines. Available: https://www.securingindustry.com/pharmaceuticals/pangea?vi?swoop?nets?41m?worth?of?fake?medicines/s40/a1766/#.V26Phbh942x [Accessed 16 Jun 2016].

[115] Woodstock J Securing our nation's prescription drug supply chain. Available: http://docs.house.gov/meetings/IF/IF14/20130425/100762/HHRG-113-IF14-Wstate-WoodcockJ-20130425.pdf [Accessed 12 Jan 2017].

[116] Dominican Today Health authorities seize counterfeit medicines in Santiago.. Available: http://www.dominicantoday.com/dr/local/2014/7/5/52027/Health?authorities?seize?counterfeit?medicines?in?Santiago [Accessed 16 Jun 2016].

[117] Medicines and Healthcare Products Regulatory Agency UK leads the way with £15.8 million seizure in global operation targeting counterfeit and unlicensed medicines and devices. Available: https://www.gov.uk/government/news/uk-leads-the-way-with-158-million-seizure-in-global-operation-targeting-counterfeit-and-unlicensed-medicines-and-devices [Accessed 3 Nov 2016].

[118] Market C Alkem lab drops on reports of substandard quality of antidiabetic drug. Available: http://www.businessstandard.com/article/newscm/alkemlabdropsonreportsofsubstandardqualityofantidiabeticdrug116060900512_1.html [Accessed 16 Jun 2016].

[119] Food and Drug Administration Philippines Product recall of specific batches of lixisenatide (Lyxumia) solution for injection (S.C.). Available: http://www.fda.gov.ph/attachments/article/299968/FDA Advisory No. 2016-002.pdf [Accessed 16 Jun 2016].

[120] LifeScan Counterfeit test strips discovered in United States: OneTouch® Ultra® and OneTouch® Basic®/Profile® test strips. Available: http://www.lifescan.com/about-us/news/149 [Accessed 18 Apr 2017].

[121] Cheng MM Is the drugstore safe? counterfeit diabetes products on the shelves. J Diabetes Sci Technol 2009;3:1516–20. 10.1177/193229680900300634 20144408 PMC2787054

[122] LifeScan Imitation OneTouch® Ultra® and OneTouch® Basic®/Profile® test strips discovered in Egypt. Available: http://www.lifescan.com/responsibility/counterfeit--products/counterfeit-alerts/egypt [Accessed 18 Apr 2017].

[123] LifeScan Counterfeit OneTouch® Ultra® blood glucose test strips discovered in Greece. Available: http://www.lifescan.com/responsibility/counterfeit-products/counterfeit-alerts/greece [Accessed 18 Apr 2017].

[124] LifeScan Counterfeit OneTouch® Ultra® blood glucose test strips discovered in Bangladesh. Available: http://www.lifescan.com/responsibility/counterfeit-products/counterfeit-alerts/bangladesh [Accessed 18 Apr 2017].

[125] LifeScan Counterfeit OneTouch® Ultra® and OneTouch® Horizon® blood glucose test strips discovered in India. Available: http://www.lifescan.com/responsibility/counterfeit-products/counterfeit-alerts/india [Accessed 18 Apr 2017].

[126] SafeMedicines Counterfeit diabetes treatments show up all over the globe. Available: http://www.safemedicines.org/counterfeit-diabetes-treatments-show-up-all-over-the-globe [Accessed 16 Jun 2016].

