# Supplementary material for: Quality of medical products for diabetes management: a systematic review
Source: BMJ Glob Health. 2019 Sep 24;4(5):e001636. doi: 10.1136/bmjgh-2019-001636 (PMC6768360; doi:10.1136/bmjgh-2019-001636)
Supplement: Supplementary data [file bmjgh-2019-001636supp001.pdf]

Appendix 1. Detailed search strategy

A list of active pharmaceutical ingredients was compiled after reviewing the British National Formulary (BNF), Monthly Index of Medical Specialities (MIMS), and MIMS online Vietnam.[1-3] The search terms were modified in concordance to each search engine or website requirement.

Pubmed:

1. diabetes AND (“medicine quality” OR “drug quality” OR falsified OR counterfeit* OR spurious OR substandard OR sub-standard OR fake OR bogus OR quack OR impostor OR rogue OR "falsely labelled" OR "falsely labeled" OR degraded OR adulterat* OR contaminat* OR dilut*)

2. (antidiabetic OR hypoglycemic OR hypoglycaemic OR “glucose lowering” OR insulin OR lispro OR protamine OR aspart OR glargine OR detemir OR degludec OR glulisine OR isophane OR NPH OR “inhaled insulin” OR sulphonylurea OR sulfonylurea OR meglitinide OR chlorpropamide OR tolbutamide OR gliclazide OR glibenclamide OR glimepiride OR glipizide OR repaglinide OR metformin OR thiazolidinedione OR pioglitazone OR acarbose OR miglitol OR voglibose OR exenatide OR liraglutide OR sitagliptin OR vildagliptin OR saxagliptin OR pramlintide OR rosiglitazone OR empagliflozin OR canagliflozin OR dapagliflozin OR dulaglutide OR alogliptin OR glyburide OR nateglinide OR colesevelam OR bromocriptine OR albiglutide OR lixisenatide OR lixinatide OR buformin OR glibornuride OR gliquidone OR mitiglinide OR miglitol OR tolazamide) AND (“medicine quality” OR “drug quality” OR falsified OR counterfeit* OR spurious OR substandard OR sub-standard OR fake OR bogus OR quack OR impostor OR rogue OR degraded OR adulterat* OR contaminat* OR dilut*)

3. glucose AND (strip OR meter OR glucometer) AND (quality OR falsified OR counterfeit OR substandard OR sub-standard OR fake OR degraded)

Embase:

1. diabetes AND (medicine quality OR drug quality OR falsified OR counterfeit* OR spurious OR substandard OR sub-standard OR fake OR bogus OR quack OR impostor OR rogue OR falsely labelled OR falsely labeled OR degraded OR adulterat* OR contaminat* OR dilut*) in basic search

2. (insulin OR lispro OR protamine OR aspart OR glargine OR detemir OR degludec OR glulisine OR isophane OR NPH OR inhaled insulin OR sulphonylurea OR sulfonylurea OR meglitinide OR chlorpropamide OR tolbutamide OR gliclazide OR glibenclamide OR glimepiride OR glipizide OR repaglinide OR metformin OR thiazolidinedione OR pioglitazone OR acarbose OR miglitol OR voglibose OR exenatide OR liraglutide OR sitagliptin OR vildagliptin OR saxagliptin OR pramlintide OR rosiglitazone OR empagliflozin OR canagliflozin OR dapagliflozin OR dulaglutide OR alogliptin OR glyburide OR nateglinide OR colesevelam OR bromocriptine OR albiglutide OR lixisenatide OR buformin OR glibornuride OR gliquidone OR mitiglinide OR miglitol OR tolazamide) ADJ5 (quality OR falsified OR counterfeit* OR spurious OR substandard OR sub-standard OR fake OR bogus OR quack OR impostor OR rogue OR falsely labelled OR falsely labeled OR degraded OR adulterat* OR contaminat* OR dilut*) in advanced search

3. glucose and (strip or meter or glucometer) and (quality or falsified or counterfeit or substandard or sub-standard or fake or degraded) {No Related Terms}
   basic search

Google Scholar (English):

1. diabetes AND (“medicine quality” OR “drug quality” OR falsified OR counterfeit OR substandard OR sub-standard OR fake OR degraded OR adulterated OR contaminated OR diluted)

2. (antidiabetic OR hypoglycemic OR hypoglycaemic OR “glucose lowering”) AND (“medicine quality” OR “drug quality” OR falsified OR counterfeit OR substandard OR sub-standard OR fake OR degraded OR adulterated OR contaminated OR diluted)
3. insulin AND (“medicine quality” OR “drug quality” OR falsified OR counterfeit OR substandard OR sub-standard OR fake OR degraded OR adulterated OR contaminated OR diluted)
4. (sulphonylurea OR sulfonylurea OR meglitinide OR chlorpropamide) AND (“medicine quality” OR “drug quality” OR falsified OR counterfeit OR substandard OR sub-standard OR fake OR degraded OR adulterated OR contaminated OR diluted)
5. (tolbutamide OR gliclazide OR glibenclamide OR glimepiride) AND (“medicine quality” OR “drug quality” OR falsified OR counterfeit OR substandard OR sub-standard OR fake OR degraded OR adulterated OR contaminated OR diluted)
6. (glipizide OR glibornuride OR gliquidone OR tolazamide OR glyburide) AND (“medicine quality” OR “drug quality” OR falsified OR counterfeit OR substandard OR sub-standard OR fake OR degraded OR adulterated OR contaminated OR diluted)
7. (metformin OR buformin OR phenformin) AND (“medicine quality” OR “drug quality” OR falsified OR counterfeit OR substandard OR sub-standard OR fake OR degraded OR adulterated OR contaminated OR diluted)
8. (repaglinide OR nateglinide OR mitiglinide) AND (“medicine quality” OR “drug quality” OR falsified OR counterfeit OR substandard OR sub-standard OR fake OR degraded OR adulterated OR contaminated OR diluted)
9. (exenatide OR albiglutide OR lixisenatide OR lixinatide) AND (“medicine quality” OR “drug quality” OR falsified OR counterfeit OR substandard OR sub-standard OR fake OR degraded OR adulterated OR contaminated OR diluted)
10. (liraglutide OR pramlintide OR dulaglutide) AND (“medicine quality” OR “drug quality” OR falsified OR counterfeit OR substandard OR sub-standard OR fake OR degraded OR adulterated OR contaminated OR diluted)
11. (pioglitazone OR rosiglitazone) AND (“medicine quality” OR “drug quality” OR falsified OR counterfeit OR substandard OR sub-standard OR fake OR degraded OR adulterated OR contaminated OR diluted)
12. (sitagliptin OR vildagliptin OR saxagliptin OR alogliptin) AND (“medicine quality” OR “drug quality” OR falsified OR counterfeit OR substandard OR sub-standard OR fake OR degraded OR adulterated OR contaminated OR diluted)
13. (acarbose OR miglitol OR voglibose) AND (“medicine quality” OR “drug quality” OR falsified OR counterfeit OR substandard OR sub-standard OR fake OR degraded OR adulterated OR contaminated OR diluted)
14. (empagliflozin OR canagliflozin OR dapagliflozin) AND (“medicine quality” OR “drug quality” OR falsified OR counterfeit OR substandard OR sub-standard OR fake OR degraded OR adulterated OR contaminated OR diluted)
15. (colesevelam OR bromocriptine OR thiazolidinedione) AND (“medicine quality” OR “drug quality” OR falsified OR counterfeit OR substandard OR sub-standard OR fake OR degraded OR adulterated OR contaminated OR diluted)
16. glucose AND (strip OR meter OR glucometer) AND (quality OR falsified OR counterfeit OR substandard OR sub-standard OR fake OR degraded)

Google Scholar (French):

1. diabète AND (“qualité du médicament” OR falsifié OR contrefaçon OR contrefait OR sous-standard OR faux OR degradé OR adultéré OR contaminé)

2. (antidiabétique OR hypoglycémique OR hypoglycémiant) AND (“qualité du médicament” OR falsifié OR contrefaçon OR contrefait OR sous-standard OR faux OR degradé OR adultéré OR contaminé)

3. (insulin) OR (“qualité du médicament” OR falsifié OR contrefaçon OR contrefait OR sous-standard OR faux OR degradé OR adultéré OR contaminé)

4. (sulphonylurée OR sulfonylurée OR “sulfamide hypoglycémiant” OR méglitinide OR chlorpropamide) AND (“qualité du médicament” OR falsifié OR contrefaçon OR contrefait OR sous-standard OR faux OR degradé OR adultéré OR contaminé)

5. (tolbutamide OR gliclazide OR glibenclamide OR glimépiride) AND (“qualité du médicament” OR falsifié OR contrefaçon OR contrefait OR sous-standard OR faux OR degradé OR adultéré OR contaminé)

6. (glipizide OR glibornuride OR gliquidone OR tolazamide OR glyburide) AND (“qualité du médicament” OR falsifié OR contrefaçon OR contrefait OR sous-standard OR faux OR degradé OR adultéré OR contaminé)

7. (metformine OR buformine OR phenformine) AND (“qualité du médicament” OR falsifié OR contrefaçon OR contrefait OR sous-standard OR faux OR degradé OR adultéré OR contaminé)

8. (répaglinide OR natéglinide OR mitiglinide) AND (“qualité du médicament” OR falsifié OR contrefaçon OR contrefait OR sous-standard OR faux OR degradé OR adultéré OR contaminé)

9. (exénatide OR albiglutide OR lixisénatide OR lixinatide) AND (“qualité du médicament” OR falsifié OR contrefaçon OR contrefait OR sous-standard OR faux OR degradé OR adultéré OR contaminé)

10. (liraglutide OR pramlintide OR dulaglutide) AND (“qualité du médicament” OR falsifié OR contrefaçon OR contrefait OR sous-standard OR faux OR degradé OR adultéré OR contaminé)

11. (pioglitazone OR rosiglitazone) AND (“qualité du médicament” OR falsifié OR contrefaçon OR contrefait OR sous-standard OR faux OR degradé OR adultéré OR contaminé)

12. (sitagliptine OR vildagliptine OR saxagliptine OR alogliptine) AND (“qualité du médicament” OR falsifié OR contrefaçon OR contrefait OR sous-standard OR faux OR degradé OR adultéré OR contaminé)

13. (acarbose OR miglitol OR voglibose) AND (“qualité du médicament” OR falsifié OR contrefaçon OR contrefait OR sous-standard OR faux OR degradé OR adultéré OR contaminé)

14. (empagliflozine OR canagliflozine OR dapagliflozine) AND (“qualité du médicament” OR falsifié OR contrefaçon OR contrefait OR sous-standard OR faux OR degradé OR adultéré OR contaminé)

15. (colesevelam OR bromocriptine OR thiazolidinedione) AND (“qualité du médicament” OR falsifié OR contrefaçon OR contrefait OR sous-standard OR faux OR degradé OR adultéré OR contaminé)

16. (glucose OR glycémie) AND (bandelette OR lecteur OR glucomètre) AND (qualité OR falsifié OR contrefaçon OR contrefait OR sous-standard OR faux OR dégradé)

Google (English):

1. (medicine OR drug OR medication) AND (diabetes OR antidiabetic OR hypoglycemic OR hypoglycaemic OR “glucose lowering”) AND (“medicine quality” OR falsified OR counterfeit OR substandard OR sub-standard OR fake OR degraded OR adulterated OR contaminated)

2. (insulin OR sulphonylurea OR sulfonylurea OR meglitinide OR chlorpropamide OR tolbutamide OR gliclazide OR glibenclamide OR glimepiride OR glipizide OR repaglinide OR metformin OR thiazolidinedione OR pioglitazone OR acarbose OR miglitol OR voglibose OR exenatide OR liraglutide OR sitagliptin) AND (quality OR falsified OR counterfeit OR substandard OR sub-standard OR fake OR degraded OR adulterated OR contaminated)

3. (vildagliptin OR saxagliptin OR pramlintide OR rosiglitazone OR empagliflozin OR canagliflozin OR dapagliflozin OR dulaglutide OR alogliptin OR glyburide OR nateglinide OR colesevelam OR bromocriptine) AND (quality OR falsified OR counterfeit OR substandard OR sub-standard OR fake OR degraded OR adulterated OR contaminated)

4. albiglutide OR lixisenatide OR lixinatide OR buformin OR glibornuride OR gliquidone OR mitiglinide OR miglitol OR tolazamide OR phenformin) AND (quality OR falsified OR counterfeit OR substandard OR sub-standard OR fake OR degraded OR adulterated OR contaminated)

5. glucose AND (strip OR meter OR glucometer) AND (quality OR falsified OR counterfeit OR substandard OR sub-standard OR fake OR degraded)

Google (French):

1. (médicament OR médication) AND (diabète OR antidiabétique OR hypoglycémiant) AND (“qualité du médicament” OR falsifié OR contrefaçon OR contrefait OR sous-standard OR faux OR degradé OR adultéré OR contaminé)

2. (insulin OR sulphonylurée OR sulfonylurée OR “sulfamide hypoglycémiant” OR méglitinide OR chlorpropamide OR tolbutamide OR gliclazide OR glibenclamide OR glimepiride OR glipizide OR glibornuride OR gliquidone OR tolazamide OR glyburide) AND (“qualité du médicament” OR falsifié OR contrefaçon OR contrefait OR sous-standard OR faux OR degradé OR adultéré OR contaminé)

3. (metformine OR buformine OR phenformine OR répaglinide OR natéglinide OR mitiglinide OR exénatide OR albiglutide OR lixisénatide OR lixinatide OR liraglutide OR pramlintide OR dulaglutide) AND (“qualité du médicament” OR falsifié OR contrefaçon OR contrefait OR sous-standard OR faux OR degradé OR adultéré OR contaminé)

4. pioglitazone OR rosiglitazone OR sitagliptine OR vildagliptine OR saxagliptine OR alogliptine OR acarbose OR miglitol OR voglibose OR empaglifozine OR canagliflozine OR dapagliflozine OR colesevelam OR bromocriptine OR thiazolidinedione) AND (“qualité du médicament” OR falsifié OR contrefaçon OR contrefait OR sous-standard OR faux OR degradé OR adultéré OR contaminé)

5. (glucose OR glycémie) AND (bandelette OR lecteur OR glucomètre) AND (qualité OR falsifié OR contrefaçon OR contrefait OR sous-standard OR faux OR dégradé)

Other websites:

Since we could not use complex search string in most of the websites, the search was conducted using Google site:search function. The combination of the search terms ‘diabetes’, ‘antidiabetics’, the names of active pharmaceutical ingredients (e.g. ‘insulin’, ‘metformin’, ‘glibenclamide’), ‘glucose meter’, ‘strip’; combined with terms relevant to medicine quality (e.g. ‘falsified’, ‘counterfeit’, ‘substandard’, ‘degraded’) were used. Below is the list of websites searched:

- Fraud.org: https://www.fraud.org/
- UK Medicines and Healthcare products Regulatory Agency: https://www.gov.uk/government/organisations/medicines-and-healthcare-products-regulatory-agency
- Agence Nationale de Sécurité du Médicament: https://ansm.sante.fr/
- Comité National Anti-Contrefaçon: http://www.cnac-contrefacon.fr/
- Centres for Disease Control and Prevention: https://www.cdc.gov/
- Health Sciences Authority: https://www.hsa.gov.sg/content/hsa/en.html
- Food and Drug Administration (FDA) Thailand: http://www.fda.moph.go.th/sites/fda_en/Pages/Main.aspx
- National Agency for Food and Drug Administration and Control (NAFDAC): https://www.nafdac.gov.ng/
- Pharmacy and Poisons Board: https://pharmacyboardkenya.org/
- Food and Drug Administration (FDA) Ghana: https://fdaghana.gov.gh/
- Food and Drug Administration (US FDA): https://www.fda.gov/drugs

References:

1. Jordan B, Martin J, Ryan R, Wagle S (eds.) *British National Formulary*. 66th ed. London: BMJ Group and Pharmaceutical Press; 2013.
2. MIMS. *MIMS Thailand: Search result for "anti-diabetic agents."* Available from: http://www.mims.com/thailand/drug/search?q=anti-diabetic+agents [Accessed 29 April 2016].
3. MIMS. *MIMS Vietnam: Search result for "anti-diabetic agents."* Available from: http://china.mims.com/vietnam/drug/search?q=anti-diabetic agents [Accessed 29 April 2016].
