# Supplementary material for: Quality of medical products for diabetes management: a systematic review
Source: BMJ Glob Health. 2019 Sep 24;4(5):e001636. doi: 10.1136/bmjgh-2019-001636 (PMC6768360; doi:10.1136/bmjgh-2019-001636)
Supplement: Supplementary data [file bmjgh-2019-001636supp003.pdf]

Appendix 3. Characteristics of prevalence surveys and equivalence studies of antidiabetic medicines

Reference	Active pharmaceutical ingredient (API)	Brand name	Outlet	Reference standard used	Test performed and analytical technique used	Total number of samples collected	Failed samples n (%)
**Prevalence surveys**							
Blume, 1993 [39]	Glibenclamide	Daonil, Diabemin, Euglucon, Pira, Glimel, Dia-Eptal, Gewaglucon, Glibenclamid, Glucobene, Normoglucon, Semi-Euglucon, Glyburide, Albert Glyberide, Diabeta, Glibenklamidum Betanas, Maninil 5, Hexaglucon, Gilemid, Azuglucon, Bastiverit, Dia-basan, Duraglucon N, Euglucon, Gliben-Puren N, Glibenclamid 3.5 GDS, Glibenclamid 3.5 Riker, Glibenclamid	Retail pharmacy, wholesaler, manufacturer	British Pharmacopeia (BP) and European standard	Active pharmaceutical ingredients (API) identity test, purity test, API content assay, uniformity of content test<br><br>Thin-layer chromatography (TLC), spectrophotometry or high performance liquid chromatography (HPLC)	187	8 (4.3%)

Reference	Active pharmaceutical ingredient (API)	Brand name	Outlet	Reference standard used	Test performed and analytical technique used	Total number of samples collected	Failed samples n (%)
		ratiopharm, Glimidstada, Gluconorm, Gluco-Tablinen, Glukoreduct, Glukovital 3.5, Glycolande N, Orabetic, Praeciglucon, Deroctyl, Euglykon, Euglucon 5, Glibenhexal, Glibenclamidum, Hemi-Daonil, Norglicem 5, Gliben, Micronase, some unknown					
Westenberger, 2005 [40]	Metformin	Unknown	Ordered from websites	US Pharmacopeia (USP)	Dissolution test, weight uniformity, API content assay and purity<br><br>HPLC, near infrared spectroscopy (NIR), NIR imaging and thermogravimetric analysis (TGA)	4	0 (0.0%)
Central Drugs Standard Control Organisation, 2009 [41]	Metformin, gliclazide, glimepiride	Glyciphage, Glycomet, Gluformin, Glizide-80, Glycigon, Reclide, Glimiprex 2, Amaryl 2, Glyree-2	Pharmacy[a]	Manufacturer standard	API identification and content assay	45	0 (0.0%)

Reference	Active pharmaceutical ingredient (API)	Brand name	Outlet	Reference standard used	Test performed and analytical technique used	Total number of samples collected	Failed samples n (%)
Ebenezer, 2015 [42]	Metformin	Glucophage, some unknown	Pharmacy and open market trader	BP	API identification and content assay<br><br>Near-infrared (NIR) spectroscopy, HPLC	179	7 (3.9%)
Islam, 2017 [43]	Metformin, glibenclamide	Unknown	Pharmacy, Depot-A, Depot-B, wholesalers (licensed)[b]	BP and USP	Dissolution test, content uniformity, API content assay<br><br>HPLC	112	21 (18.8%)*
**Equivalence studies**							
Attorrese, 2007 [44]	Glimepiride	Adiamyl, Amadiab-2, Bioglic, Diagril, Diabold, Diameprid, Dolcyl, Evopride, Hanall glimepiride, Geliemeiniao Jiaonang, GLA-DM, Glimepibal, Glimax, Glimepiride (Boryung), Glimepiride (Hanni), Gimepirida, Glimepirida (Esterlina), Glimepirida (Eurofarma), Glimulin-2, Glusafe, Metrix,	Unknown	Manufacturer specification of innovator brand Amaryl	API content assay, impurities, residual solvents, dissolution test<br><br>HPLC, gas chromatography	23	13 (56.5%)

Reference	Active pharmaceutical ingredient (API)	Brand name	Outlet	Reference standard used	Test performed and analytical technique used	Total number of samples collected	Failed samples n (%)
		Panabutol, Taboss					
Hamdan, 2010 [45]	Metformin	Glucophage, Glymet, Metforal, Diaphage, Formit	Unknown	BP	Dissolution test, API content identification and assay<br><br>UV-spectrophotometer, Fourier-transform infrared (FTIR) spectroscopy	5	1 (20.0%)
Chandrasekaran, 2011 [46]	Metformin	Unknown	Unknown	Not specified	Dissolution test<br><br>UV-visible spectrophotometry	5	0 (0.0%)
Afifi, 2012 [47]	Metformin	Glucophage, Formit, Glucare, Dialon, Metaphage, Metfor	Pharmacy[a]	USP	API content assay, dissolution test, disintegration test, weight uniformity, hardness test, friability test	6	0 (0.0%)
Chatur, 2012 [48]	Voglibose	Voglitor MD, Vobit MD, Prandial, Obligo MD	Pharmacy[a]	Not specified	Weight uniformity, hardness test, friability test, disintegration test, content uniformity, wetting time and water absorption, USP in-vitro dissolution test<br><br>UV-spectrophotometry	5	1 (20.0%)
Olusola, 2012 [49]	Metformin	Unknown	Registered pharmacy	BP	Disintegration test, weight uniformity, hardness test, friability test, BP 2007 content assay<br><br>UV-visible spectrophotometry	8	1 (12.5%)
Oyetunde, 2012 [50]	Metformin	Glucophage, some unknown	Country representative seller and wholesaler	BP	API content, dissolution test<br><br>UV-visible spectrophotometer	5	2 (40.0%)

Reference	Active pharmaceutical ingredient (API)	Brand name	Outlet	Reference standard used	Test performed and analytical technique used	Total number of samples collected	Failed samples n (%)
El-Sabawi, 2013 [51]	Glibenclamide	Daonil, Melix, Glunil, Glibil, Glibemide, Glucomid, Glucophage, Glymet, Metforal, Diaphage, Formit	Community pharmacy	BP and USP	API content assay, impurities, friability test, dissolution test HPLC, TLC	6	2 (33.3%)
Labu, 2013 [52]	Metformin	Unknown	Pharmacy[a]	BP and USP	Dissolution test, disintegration test, weight uniformity, hardness test, friability test, thickness test, API content assay<br><br>Titrimetry, UV-visible spectrophotometer	7	0 (0.0%)
Ajala, 2014 [53]	Metformin	Unknown	Community pharmacy	BP and USP	Weight uniformity, crushing strength, friability, disintegration, dissolution, API content assay	8	3 (37.5%)
Betari, 2014 [54]	Sitagliptin	Unknown	Unknown	USP and Food and Drug Administration (FDA)	Weight uniformity, hardness test, friability test, content uniformity, dissolution test<br><br>UV-visible spectrophotometry, UV spectrophotometry	5	0 (0.00%)
Elango, 2014 [55]	Metformin	Unknown	Pharmacy[a]	Not specified	Physical inspection (including size and weight), purity test, friability test, API content assay, dissolution test<br><br>UV-spectrophotometry	15	3 (20.0%)
Elhamili, 2014 [56]	Glibenclamide	Gliboral, Glynase, Glib-5	Private pharmacy	BP	Dissolution test, disintegration test, friability test, identification and API content assay	3	0 (0.0%)

**Reference**	**Active pharmaceutical ingredient (API)**	**Brand name**	**Outlet**	**Reference standard used**	**Test performed and analytical technique used**	**Total number of samples collected**	**Failed samples n (%)**
					HPLC-UV, infrared (IR) spectroscopy		
Abdulhameed, 2016 [57]	Metformin	Unknown	Manufacturing company	USP	API content assay, friability test, dissolution test<br><br>UV-visible spectrophotometry	5	0 (0.0%)
Gupta, 2016 [58]	Metformin	Unknown	Hospital pharmacy and local private pharmacy	USP	Weight uniformity, friability test, disintegration test, dissolution test, API content assay<br><br>UV-visible spectrophotometry	4	0 (0.0%)
Sachan, 2016 [59]	Meftormin	Melmet, Obimet, Glycomet, Glyciphage	Unknown	Indian Pharmacopeia	Visual inspection, uniformity mass test, hardness test, friability test, disintegration test, API content assay, dissolution test<br><br>UV spectrophotometry, UV-visible double beam spectrophotometry	4	0 (0.0%)
Sakr, 2016 [60]	Glibenclamide	Glymide, Doabetic, Glibil, Daonil,	Community pharmacy	BP and USP	Dissolution test, weight uniformity, friability test, content uniformity, disintegration time<br><br>HPLC, TLC	8	0 (0.0%)
Alam, 2017 [61]	Glibenclamide	Daonil, Glibil, Doabetic, Diatab, Glymide	Retail pharmacy	USP	Friability test, hardness test, weight uniformity, disintegration test, content uniformity<br><br>UV-spectrophotometry, UV-visible spectrophotometry	5	0 (0.0%)
Eraga, 2017 [62]	Metformin	Unknown	Pharmacy[a]	BP	Hardness test, friability test, in vitro drug release, dissolution test, API	10	8 (80.0%)**

Reference	Active pharmaceutical ingredient (API)	Brand name	Outlet	Reference standard used	Test performed and analytical technique used	Total number of samples collected	Failed samples n (%)
					content assay, weight uniformity, disintegration test<br><br>UV-spectrophotometry, reverse phase (RP)-HPLC		
Aivalli, 2018 [63]	Metformin, glibenclamide	Unknown	District hospital, private pharmacy, market	Not specified	API identification test, API content assay, uniformity of content test , weight uniformity, dissolution test<br><br>HPLC, IR spectroscopy	10	0 (0.0%)

Note:

If unspecified, this means that in the source document the details (e.g. size, public/private, registered/not) on the pharmacies sampled were not described.

*In Islam 2017,[44] only the number of medicine failing each quality test was mentioned. Since one medicine may fail more than one test, the failure rate was recorded as the highest possible number of samples failing one of the tests.

**In Eraga 2017,[62] uniformity of content was assessed using two methods i.e. UV spectrophotometry and reverse-phase high performance liquid chromatography. There are several discrepancies in the results of these two tests. Therefore, if samples failed either, they will be categorised as failed samples.

[a]The type of pharmacy (private or public) was not specified.

[b]Depot-A: "a site having a pharmacist with at least three years' pharmacy training", Depot-B: " a site having a doctor or retired nurse in attendance."
