# Supplementary material for: Quality of medical products for diabetes management: a systematic review
Source: BMJ Glob Health. 2019 Sep 24;4(5):e001636. doi: 10.1136/bmjgh-2019-001636 (PMC6768360; doi:10.1136/bmjgh-2019-001636)
Supplement: Supplementary data [file bmjgh-2019-001636supp004.pdf]

Number of failing samples per type of defect and API in the prevalence surveys (the % are calculated out of the total number of failing samples, N= 36)

Because of the limited number of samples tested for quality in the studies included in this review, the figures should not be interpreted as representative of the prevalence of specific SF antidiabetics (please refer to the discussion section of the current paper for more details)

Quality component	Metformin n (%)	Glibenclamide n (%)	Total N (%)
Assay/content	12 (33.3%)	14 (38.9%)	26 (72.2%)
Uniformity of content	0 (0.0%)	11 (30.6 %)	11 (30.6%)
Dissolution	2 (5.6 %)	5 (13.9 %)	7 (19.4%)

Note: Not all samples were subject to pharmaceutical quality tests. One sample may have one or more defect, hence the total number used as the denominator in this table does not represent the total count of failing samples.

Number of failing samples per type of defect and API in the equivalence studies (the % are calculated out of the total number of failing samples, N= 37)

Because of the limited number of samples tested for quality in the studies included in this review, the figures should not be interpreted as representative of the prevalence of specific SF antidiabetics (please refer to the discussion section of the current paper for more details)

Quality component	Metformin n (%)	Glibenclamide n (%)	Glimepiride n (%)	Sitagliptin n (%)	Voglibose n (%)	Total N (%)
Dissolution/ disintegration/drug release	5 (13.5%)	0 (0.0%)	12 (32.4%)	2 (5.4%)	1 (2.7%)	20 (54.1%)
Purity/contamination /residue	3 (8.1%)	0 (0.0%)	3 (8.3%)	0 (0.0%)	0 (0.0%)	6 (16.2%)
Assay/content	10 (27.0%)**	3 (8.1%)	1 (2.7%)	0 (0.0%)	0 (0.0%)	14 (37.8%)
Friability*	4 (11.1%)	0 (0.0%)	0 (0.0%)	0 (.0%)	0 (0.0%)	4 (11.1%)
Weight	1 (2.7%)	0 (0.0%)	0 (0.0%)	0 (0.0%)	0 (0.0%)	1 (2.7%)
Hardness	6 (16.2%)	0 (0.0%)	0 (0.0%)	0 (0.0%)	0 (0.0%)	6 (16.2%)

**Friability is defined as 'the tendency for a tablet to chip, crumble or break following compression'.*[63] *A good balance between hardness (hard enough not to break during packaging or distribution) and friability (friable enough to facilitate disintegration, dissolution, and absorption) is required for tablets.*[63]

***Attorrese 2007 did not specify how many samples failing content assay on day 0 before samples were exposed to stressed condition.*

Note: Not all samples were subject to pharmaceutical quality tests. One sample may have one or more defect, hence the total number used as the denominator (N=41) in this table does not represent the total count of failing samples.
