# Supplementary material for: Quality of medical products for diabetes management: a systematic review
Source: BMJ Glob Health. 2019 Sep 24;4(5):e001636. doi: 10.1136/bmjgh-2019-001636 (PMC6768360; doi:10.1136/bmjgh-2019-001636)
Supplement: Supplementary data [file bmjgh-2019-001636supp005.pdf]

Appendix 5. Prevalence surveys concordance with MEDQUARG items checklist

No	Item	Blume, 1993	CDSCO, 2009	Westenberger, 2014	Ebenezer, 2015	Islam, 2017
1	Title/abstract/keywords	N	N	Y	Y	N
2	Introduction	N	Y	N	Y	Y
**Methods**						
3	Survey details	N	N	N	Y	N
4	Definitions	N	N	N	Y	N
5	Outlets	N	N	N	N	N
6	Sampling design	N	Y	N	Y	N
7	Samplers	N	Y	N	Y	N
8	Statistical methods	N	N	N	Y	Y
9	Ethical issues	N	N	N	Y	N
10	Packaging	N	Y	Y	Y	Y
11	Chemical analysis	Y	N	Y	Y	N
12	Method validation	N	N	N	Y	N
13	Blinding	N	N	N	Y	N
**Results**						
14	Outlets	N	Y	N	Y	Y
15	Missing samples	N	N	N	Y	N
16	Packaging and chemistry results	N	Y	Y	Y	N
17	Category of poor-quality medicine	N	N	N	Y	N
18	State company and address as given on packaging	N	Y	N	Y	N
19	Sharing data with MRA	N	Y	N	N	Y
20	Dissemination	N	Y	N	N	N
**Discussion**						

No	Item	Blume, 1993	CDSCO, 2009	Westenberger, 2014	Ebenezer, 2015	Islam, 2017
21	Key results	Y	Y	Y	Y	Y
22	Limitations	N	N	N	Y	Y
23	Interpretation	Y	Y	N	Y	Y
24	Intervention	Y	N	N	Y	Y
**Other Information**						
25	Conflict of interest	N	N	N	N	N
26	Funding	N	N	N	N	N
	Total score	4	11	5	21	9
		15.4%	42.3%	19.2%	80.8%	34.6%

Y : Yes, N : No
