# Supplementary material for: Quality of medical products for diabetes management: a systematic review
Source: BMJ Glob Health. 2019 Sep 24;4(5):e001636. doi: 10.1136/bmjgh-2019-001636 (PMC6768360; doi:10.1136/bmjgh-2019-001636)
Supplement: Supplementary data [file bmjgh-2019-001636supp006.pdf]

Appendix 6. Other types of articles discussing antidiabetic medicine quality

No	Title	First author	Year
1	Lessons from Nigeria: the fight against counterfeit drugs in Africa [1]	Akunyili, D	2006
2	Medicines Quality Database [2]	United States Pharmacopeial Convention	2008
3	Is the drugstore safe? Counterfeit diabetes products on the shelves [3]	Cheng, MM	2009
4	The dangerous world of counterfeit prescription drugs [4]	Toscano, P	2011
5	FDA alerts companies to stop illegal sale of treatments for diabetes [5]	US Food and Drug Administration	2013
6	Counterfeit diabetes products and the ethical question of access [6]	Cheng, MM	2015
7	Media reports on medicine quality: Focusing on USAID-assisted Countries [7]	McGinnis, M	2015
8	Illegally sold diabetes treatments [8]	US Food and Drug Administration	2016
9	Beware of illegally marketed diabetes treatments [9]	US Food and Drug Administration	2016
10	International SOS warns travellers on World Diabetes Day: Pack your own medication and watch out for counterfeit drugs [10]	International SOS	Unknown
11	Counterfeit diabetes treatments show up all over the globe [11]	Partnership for Safe Medicines	Unknown

References

1. Akunyili D. Lessons from Nigeria: the fight against counterfeit drugs in Africa. *Diabetes Soc*. 2006;51(3):41-43.
2. United States Pharmacopeial Convention. *Medicines Quality Database*. Available from: http://apps.usp.org/app/worldwide/medQualityDatabase/reportResults.html?country=Ethiopia%2BGhana%2BKenya%2BMozambique%2BNigeria%2BCambodia%2BLao+PDR%2BPhilippines%2BThailand%2BViet+Nam%2BBolivia%2BColombia%2BEcuador%2BGuatemala%2BGuyana%2BPeru&period=2017 [Accessed 17 December 2018].
3. Cheng MM. Is the drugstore safe? Counterfeit diabetes products on the shelves. *Journal of Diabetes Science and Technology*. 2009;3(6):1516–20.

4. Toscano P. *The dangerous world of counterfeit prescription drugs*. Available from: http://usatoday30.usatoday.com/money/industries/health/drugs/story/2011?10?09/cnbc?drugs/50690880/1 [Accessed 16 June 2016].
5. US Food and Drug Administration. FDA alerts companies to stop illegal sale of treatments for diabetes. Available from: http://www.fda.gov/ForConsumers/ProtectYourself/HealthFraud/ucm352276.htm. [Accessed 16 June 2016].
6. Cheng MM, Gedeon C. Counterfeit diabetes products and the ethical question of access. *Diabetes Manag*. 2015;5(5):341–7.
7. McGinnis M. *Media reports on medicine quality: Focusing on USAID-assisted countries by the promoting the quality of medicines program*. Available from: https://www.usp.org/sites/default/files/usp_pdf/EN/PQM/june2015-pqm-media-reports.pdf [Accessed 1 May 2016].
8. US Food and Drug Administration. *Illegally sold diabetes treatment*. Available from: http://www.fda.gov/ForConsumers/ProtectYourself/HealthFraud/ucm352276.htm [Accessed 16 June 2016].
9. US Food and Drug Administration. *Beware of illegally marketed diabetes treatments*. Available from: http://www.fda.gov/ForConsumers/ConsumerUpdates/ucm361487. htm [Accessed 16 June 2016].
10. International SOS. *International SOS warns travellers on World Diabetes Day: Pack your own medication and watch out for counterfeit drugs.* Available from: https://www.internationalsos.com/clinicsinchina/en/872.aspx [Accessed 16 June 2016].
11. SafeMedicines. *Counterfeit diabetes treatments show up all over the globe*. Available from: http://www.safemedicines.org/counterfeit-diabetes-treatments-show-up-all-over-the-globe [Accessed 16 June 2016].
